# Confined Migration Drives Stem Cell Differentiation

**DOI:** 10.1002/advs.202415407

**Published:** 2025-05-08

**Authors:** Xu Gao, Yixuan Li, Jia Wen Nicole Lee, Jianxuan Zhou, Vaishnavi Rangaraj, Jennifer Marlena, Andrew W. Holle

**Affiliations:** ^1^ Department of Biomedical Engineering College of Design and Engineering National University of Singapore Singapore 117583 Singapore; ^2^ Mechanobiology Institute National University of Singapore Singapore 117411 Singapore

**Keywords:** confined migration, human mesenchymal stem cells, nuclear mechanosensing, stem cell differentiation, stem cell epigenetics

## Abstract

In both endogenous and exogenously‐introduced human mesenchymal stem cells (hMSCs), homing to sites of regeneration requires navigation through complex extracellular matrix environments that impose confinement on migrating cells. Despite its prevalence in vivo, the impact of confinement on hMSC differentiation remains poorly understood. To address these questions, a physiologically relevant, flow‐free polydimethylsiloxane‐based microchannel system with confining widths ranging from 3 to 10 µm in width, is developed. In these microchannel systems, it is found that hMSCs migrate faster and experience significant nuclear deformation in 3 µm wide channels compared to wider 10 µm channels. These morphological changes persist for days postexit, implying that stem cells possess a mechanical memory of their past confined migration. High degrees of nuclear deformation also correlated with substantial changes in genome regulation, as cells displayed significant H3K9 acetylation postconfinement. In these postconfinement stem cells, significantly higher expression levels of RUNX2 along with a higher degree of nuclear‐to‐cytoplasmic shuttling are found, suggesting that short confined migration can stimulate osteogenic differentiation. Finally, it is found that nuclear mechanosensing via the cytoskeleton is not the primary factor driving confinement‐induced differentiation. These results suggest that physiological confinement can serve as a key mechanical cue promoting early osteogenic differentiation in hMSCs.

## Graphical abstract

Human mesenchymal stem cell (hMSC) homing to sites of regeneration requires navigation through mechanically confining extracellular matrix environments, inducing rapid and dramatic morphological adaptation. By exposing hMSCs to specific levels of physiological confinement, it is demonstrated that confinement can induce osteogenesis, and that this fate specification occurs due to nuclear deformation, not active force transduction across the nuclear envelope.

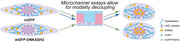

## 1 Introduction

Human mesenchymal stem/stromal cells (hMSCs), which have also been referred to as human Mesenchymal Stromal Cells (hMSCs) and human Medicinal Signaling Cells (hMSCs), have shown potential in regenerative medicine given their ease of isolation, mild immune response, low mutagenic risk, and multilineage differentiation capability.^[^ ^1^ ^]^ To enhance this potential, a thorough understanding of how these progenitor cells interact with their complex extracellular matrix (ECM) microenvironment is required. Interstitial spaces within this ECM present varying levels of confinement to stem cells as they migrate toward sites of regeneration.^[^ ^2^, ^3^, ^4^, ^5^ ^]^ While a number of studies have shed light on mechanosensitive regulators of hMSC fate,^[^ ^6^ ^]^ including matrix stiffness, shear force, tensile stress, and hydrostatic pressure, the consequences of physiological confinement on hMSC lineage commitment are not well known. Previous studies have shown that hMSCs migrating in confinement display alterations in cytoskeletal organization and undergo nuclear deformation.^[^ ^7^ ^]^ Moreover, hMSCs in osteogenic differentiation media that migrate through narrow transwell assays have been found to display enhanced bone differentiation, although this is accompanied by DNA damage.^[^ ^8^ ^]^ However, the degree to which stem cells can sense confinement and differentiate as a function of this mechanical signal alone remains unknown.

One major reason for this has been a lack of understanding of the nature of the interstitial spaces through which stem cells migrate. The rise of intravital microscopy has allowed for the quantification of interstitial spaces in various tissues, revealing that the average width of tissue tracks ranges from about 3 to 10 µm.^[^ ^9^ ^]^ Cells have also been observed to follow tissue tracks up to 750 µm in length,^[^ ^9^ ^]^ meaning that cells in vivo are exposed to sustained confinement beyond levels found in transwell assays. Therefore, various in vitro models have been developed to study confined migration,^[^ ^10^ ^]^ including micropatterned substrates, vertical confinement, grooved substrates, polydimethylsiloxane (PDMS)‐based microchannels, and patterned gels.

When cells migrate into and through these physiological spaces, both in vivo and in vitro, the nucleus bears the brunt of the resulting compression. This nuclear deformation, induced by different mechanical stimuli, has been shown to modulate cellular behaviors via a number of mechanosensitive pathways.^[^ ^11^ ^]^ One such modality is cytoskeleton‐based nuclear mechanosensing, in which traction forces generated by actomyosin contractility are transmitted to mechanosensitive adhesions comprised of the “linker of nucleoskeleton and cytoskeleton” (LINC) complex at the nuclear envelope.^[^ ^12^ ^]^ Forces transmitted to the nucleus via the LINC complex have been shown to regulate nucleocytoplasmic transport,^[^ ^13^ ^]^ chromatin organization, and epigenetic remodeling,^[^ ^14^, ^15^, ^16^, ^17^ ^]^ thereby influencing hMSC fate.^[^ ^18^, ^19^ ^]^ In contrast, deformation‐based nuclear mechanosensing occurs via simple nuclear compression, even in the absence of cytoskeletal force. This modality has been found to elicit a wide variety of mechanosensitive responses, including nuclear pore complex activity,^[^ ^20^ ^]^ nuclear ion channel activation,^[^ ^21^ ^]^ and chromatin reorganization.^[^ ^22^, ^23^ ^]^ While nuclear reorganization is a hallmark of confinement, it remains unclear how the interplay between cytoskeleton‐based and deformation‐based nuclear mechanosensing contributes to confinement‐induced changes in stem cell behavior.

Here, we have designed PDMS‐based microchannels, for which photolithography was leveraged to precisely control channel length, width, and height to allow for tight control of experimental variables. We have established a relationship between channel geometry and stem cell differentiation, showing that stem cells can differentiate as a function of the confinement to which they are exposed during migration. We have also explored the mechanism of this response by answering whether deformation‐based or cytoskeleton‐based nuclear mechanosensing drives confinement‐induced stem cell differentiation. Overall, our results suggest that migration through physiologically‐relevant confinement is sufficient to initiate differentiation pathways in adult stem cells in a nuclear deformation‐dependent process.

## 2 Results

### 2.1 Stem Cells Migrate Quickly and Undergo Major Nuclear Deformation in Extreme Confinement

**Figure 1:**
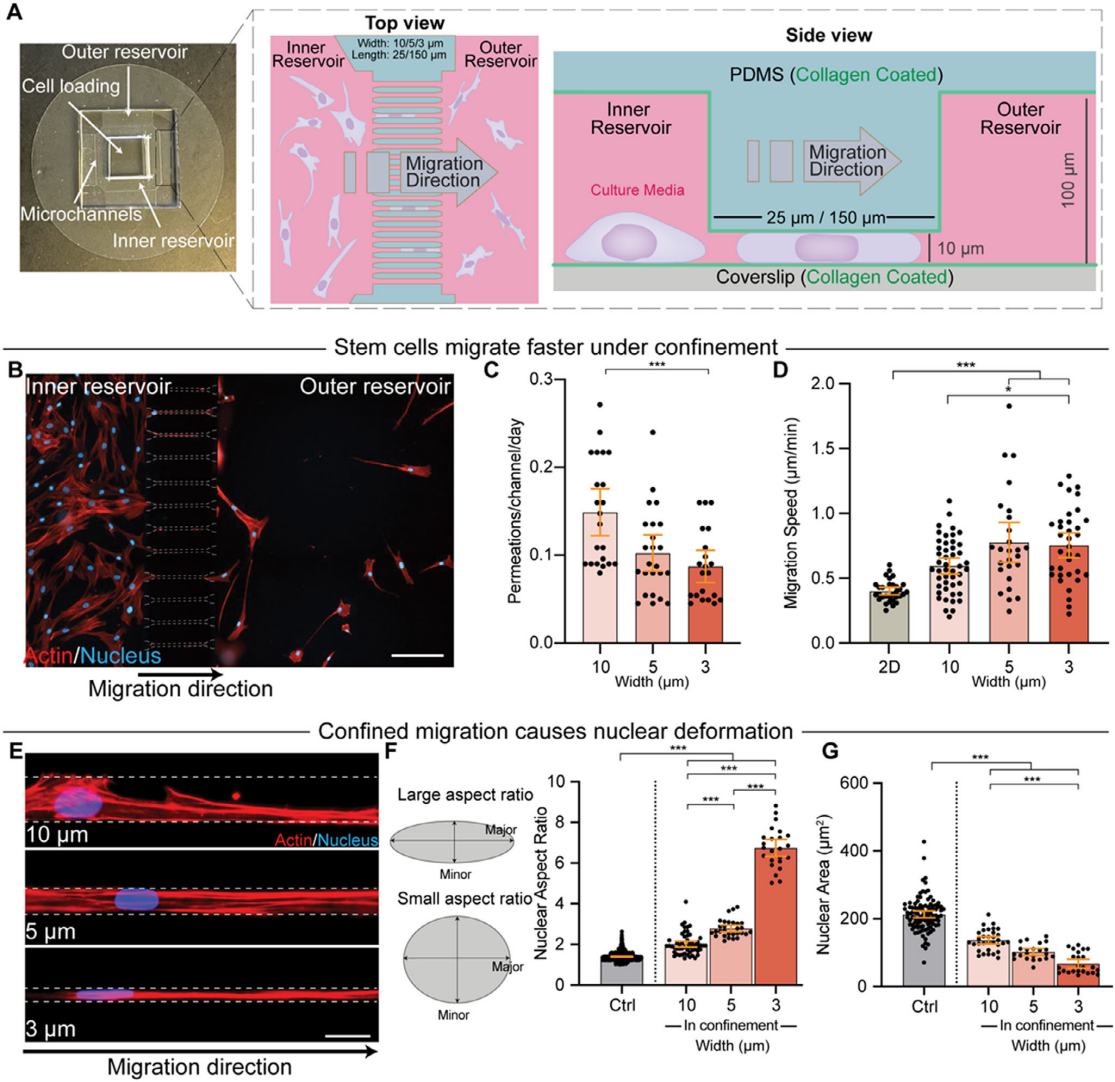
hMSCs move faster in confined microchannels while experiencing strong nuclear deformation. A) Picture (left) and schematic view (middle and right) of the in vitro confined migration system. B) Representative image demonstrating that cells can migrate through long, 3 µm‐wide microchannels without chemoattractant. White dashed lines indicate the position of microchannels. Actin, red; Nucleus, blue. Scale bar: 150 µm. C) Permeation rate (number of cells migrated per channel per day) of hMSCs in long microchannels (*n* = 22, 24, and 21). D) Average migration speed in long microchannels (*n* = 31, 47, 26, and 35). E) Representative images demonstrating nuclear deformation under different degrees of confinement. Actin, red; Nucleus, blue. Scale bar: 10 µm. F,G) Quantification of nuclear aspect ratio F) and nuclear area G) under long microchannels (*n* = 916 [100 data points were randomly selected for data presentation], 32, 22, and 24 for (F,G)). Error bars represent 95% confidence intervals. One‐way ANOVA with Bonferroni's posthoc test was applied for (C). Welch ANOVA with Dunnett's T3 posthoc test was applied for (D, F, G). *p < 0.05, ***p < 0.001.

To test the ability of hMSCs to migrate through diverse physiological confinement found in tissue tracks in vivo, we designed PDMS‐based microchannels with heights of 10 µm, lengths of either 25 µm (short) or 150 µm (long), and widths of 3 µm (narrow), 5 µm (intermediate), or 10 µm (wide), all of which were collagen‐coated (**Figure** **1**A; and Figure S1, Supporting Information). The open‐reservoir design of this chip allows for facile cell seeding and ensures a balance in hydrostatic pressure in both the inner and outer reservoirs, eliminating any confounding flow. Using live cell microscopy, we found that hMSCs are able to migrate into and through all of the microchannel designs, including the long, narrow microchannels, without the need for a chemoattractant (Figure 1B). This migration was confinement‐sensitive, as decreasing channel widths resulted in a reduction in the number of cells that fully permeated the channels (Figure 1C). However, cells that did manage to migrate through narrow (3 µm) and intermediate (5 µm) channels did so at a faster speed (Figure 1D), mirroring the mesenchymal‐to‐amoeboid transition observed in cancer cells in response to confinement.^[^ ^24^ ^]^ Fixation and staining confirmed that hMSCs exhibit major morphological changes when confined. As expected, migration into long microchannels causes an increase in nuclear aspect ratio as a function of confinement degree (Figure 1E,F). We also found that as cells enter progressively tighter microchannels, their 2D projected area drops concomitantly (Figure 1G). Daily monitoring of cell migration through the channels and into the outer reservoir showed that cells continually permeate through the chip for the duration of the experiment (Figure S2, Supporting Information).

### 2.2 Mechanical Memory in Stem Cell Morphology Postconfined Migration

**Figure 2:**
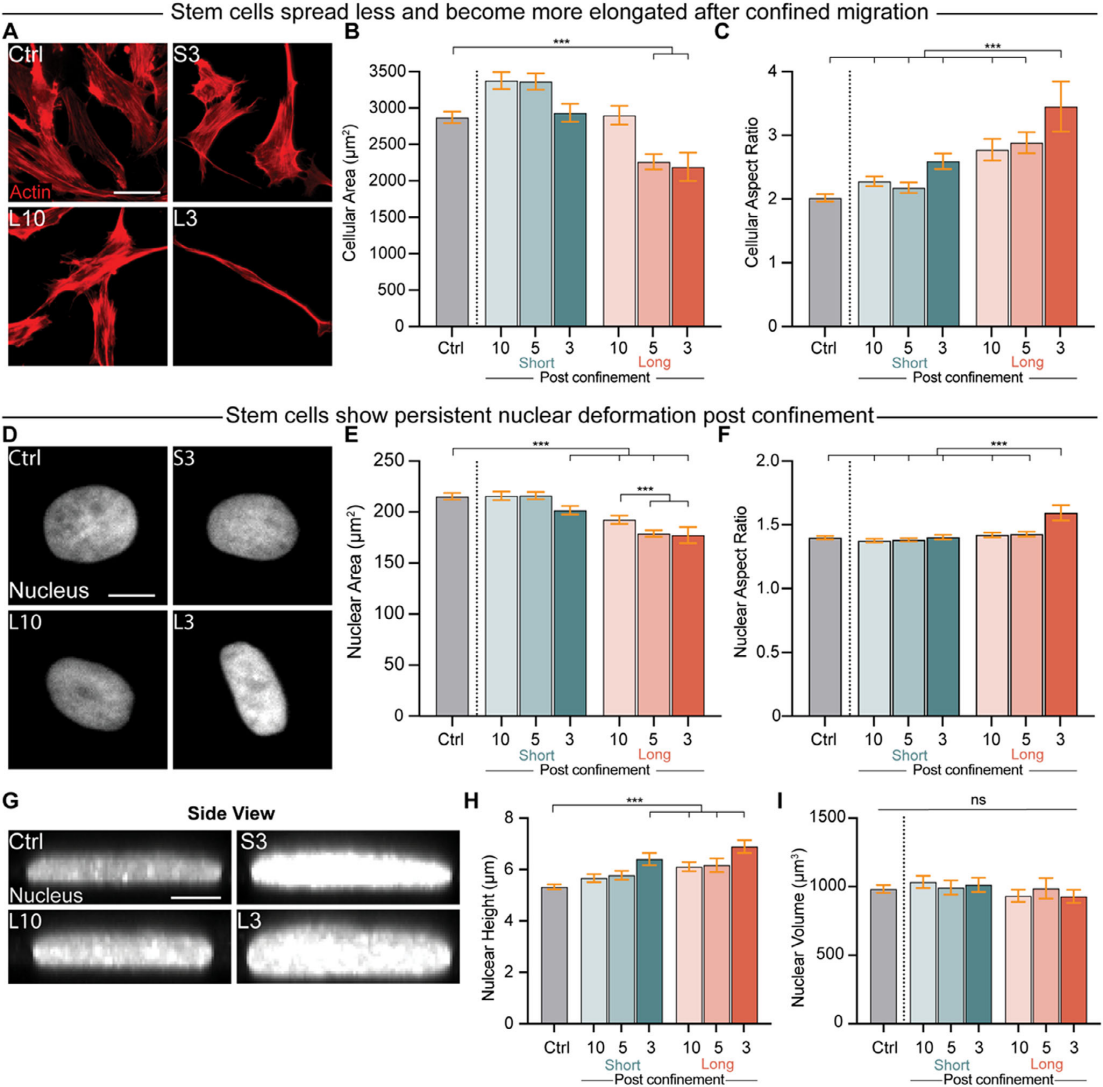
Stem cell morphology is altered after confined migration. A) Representative images demonstrating that cells became smaller after migrating through long, 3 µm‐wide microchannels on Day 4. Actin, red. Scale bar: 50 µm. B,C) Cellular area B) and cellular aspect ratio C) of cells before and after traversing short and long microchannels with different widths (*n* = 916, 679, 764, 521, 530, 490, and 157). D) Representative images demonstrating that nuclei become elongated after migrating through long, 3 µm‐wide microchannels. Nucleus, gray. Scale bar: 10 µm. E,F). Nuclear area E) and nuclear aspect ratio F) of cells before and after traversing short and long microchannels with different widths (*n* = 916, 679, 764, 521, 530, 490, and 157). G) y–z projection of confocal images showing that nuclei weretaller after migrating through long, 3 µm‐wide microchannels. Nucleus, gray. Scale bar: 5 µm. H,I) Nuclear volume H) and nuclear height I) of cells before and after traversing short and long microchannels with different widths (*n* = 281, 126, 101, 78, 112, 60, and 93). Welch ANOVA with Games–Howell's posthoc test was applied for (B, C), (E, F) and (H). One‐way ANOVA with Bonferroni's posthoc test was used for (I). Error bars represent 95% confidence intervals. ***p < 0.001. ns, no significant difference.

A key feature of this microchannel system and its segregated outer reservoirs is that it allows for the observation of postconfinement cells, with direct knowledge of which channels the cells moved through in the past. To assess how hMSCs adapt to confinement, we fixed cells after 4 days of culture and analyzed cellular and nuclear morphology as a function of confinement. Stem cell morphology was significantly altered after migration through long narrow microchannels, with cellular area dropping by roughly 25% and aspect ratio increasing by 75% compared to control cells (**Figure** **2**A–C). This response was confinement‐sensitive, as migration through long wide channels did not induce the same degree of change in area and aspect ratio. Significant increases in actin intensity and fiber anisotropy were observed postconfinement, suggesting that confinement induces enhanced stress fiber formation and a more organized cytoskeleton (Figure S3E–G, Supporting Information). Focal adhesion organization was also influenced by confinement, as paxillin staining revealed an increase in focal adhesion cluster area and intensity in cells that had navigated the tightest microchannels (Figure S3A–D, Supporting Information).

The nucleus, which was heavily deformed in narrow confinement, did not fully recover to its initial morphology after cells moved to the unconfined outer reservoir (Figure 2D). Similar to cell area, nuclear area also dropped post confinement, with the biggest effects seen in cells that had traversed long channels (Figure 2E). Changes in nuclear aspect ratio and nuclear strain were much more sensitive to confinement, as only cells that had exited long narrow channels retained their elongated shape (Figure 2F; and Figure S4, Table S1, Supporting Information). Interestingly, no significant DNA damage was observed in cells as a function of confinement dimensions, as determined by the number of γH2AX foci (Figure S5, Supporting Information). As nuclear area changes can only reflect 2D nuclear dynamics, we next looked to determine how this confining journey would affect the 3D morphology of nuclei. By measuring the height of postconfinement nuclei (Figure 2G), we found that long and tight confinement both drove increases in nuclear height after exit (Figure 2H). Interestingly, this increase in height was counterbalanced by the changes in nuclear area we had previously measured. Regardless of the microchannel profile through which the cells migrated, we found no significant changes in nuclear volume (Figure 2I), in agreement with recent works that have emphasized that nuclear volume regulation occurs independently of nuclear shape changes.^[^ ^25^ ^]^ These changes in cell morphology as a function of mechanical confinement led us to question the degree to which cells utilize mechanosensitive signaling pathways and alter their nuclear transport mechanisms.^[^ ^20^ ^]^

### 2.3 Confinement Induces Duration‐Dependent Mechanosensitive Responses

**Figure 3:**
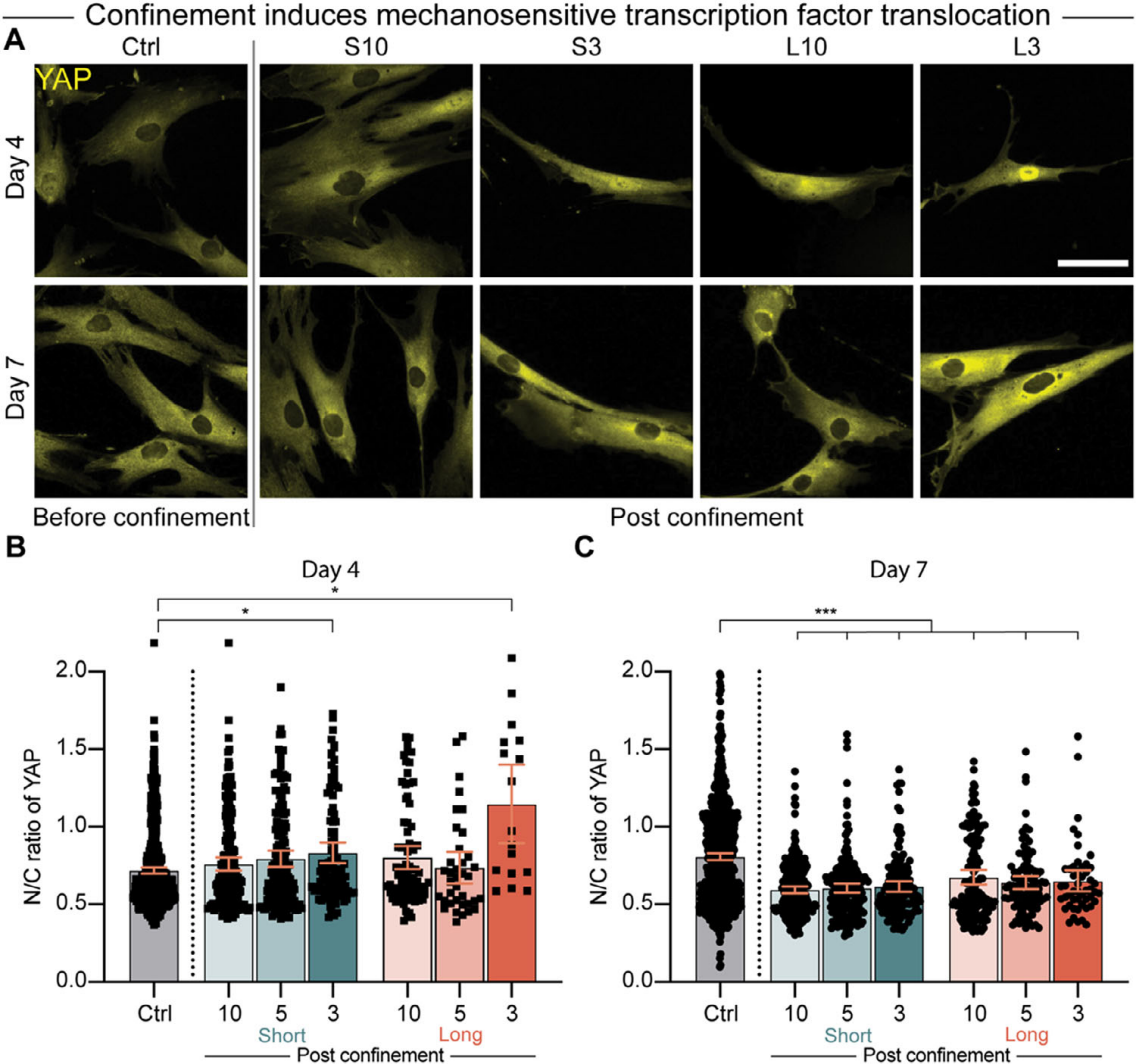
Confined migration induces mechanosensitive responses in hMSCs. A) Representative images of YAP staining in cells before and after moving out of short and long microchannels with widths of 10 and –3 µm on Day 4 and Day 7. YAP, yellow. Scale bar: 50 µm. B,C) Quantification of the N/C ratio of YAP in cells before and after migrating through different microchannels on Day 4 B) and Day 7 C). (*n* = 618, 194, 154, 102, 78, 37, and 17 for (B); *n* = 817, 229, 196, 144, 135, 95, and 50 for (C)). Welch ANOVA with Dunnett's T3 posthoc test was applied for (B) and Welch ANOVA with Games–Howell's posthoc test was applied for (C). *p < 0.05, ***p < 0.001.

To determine whether confined migration acts as a persistent mechanical cue, we measured nuclear translocation of the mechanosensitive transcription factor YAP, which has been shown to be active in hMSCs. Long, narrow confinement, which had resulted in the largest degree of morphological changes to nuclei, was found to induce a significant increase in YAP translocation after four days (**Figure** **3**A,B), in agreement with previous reports of deformation‐induced YAP activation.^[^ ^26^ ^]^ Interestingly, even though morphological changes to cells persisted (Figure S6, Supporting Information), YAP translocation was not present after 7 days, suggesting that transcription factor localization is a transient process that occurs at specific durations postconfinement (Figure 3C).

### 2.4 Confined Migration Drives Stem Cell Differentiation in a Width‐Dependent Fashion

**Figure 4:**
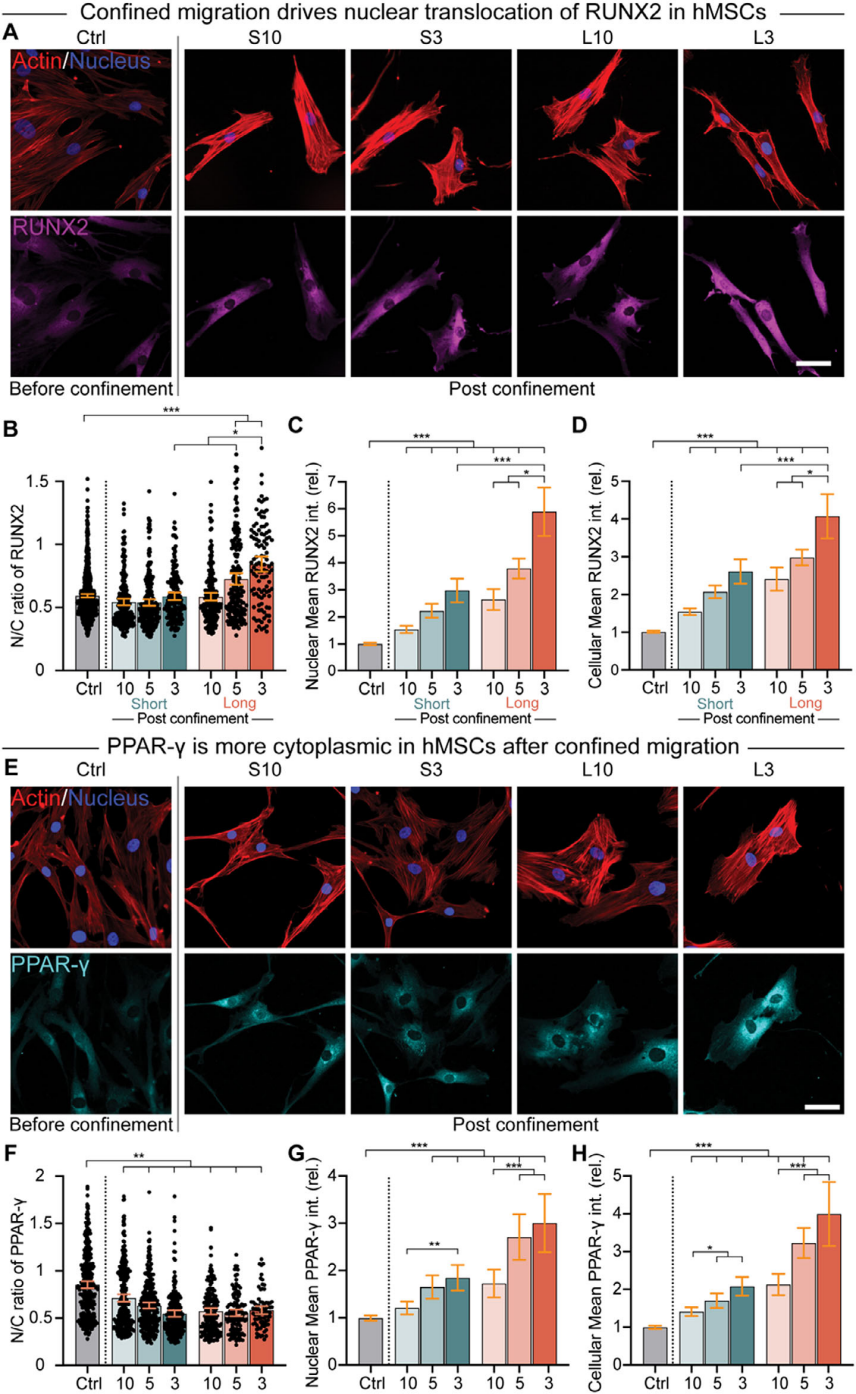
Confined migration affects stem cell lineage commitment. A) Representative images of RUNX2 staining in hMSCs before and after moving out of short and long microchannels with widths of 10 and 3 µm on Day 7. RUNX2, magenta; Actin, red; Nucleus, blue. Scale bar: 50 µm. B–D) N/C ratio B), relative nuclear mean intensity C), and relative cellular mean intensity D) of RUNX2 in hMSCs (*n* = 741, 233, 258, 170, 200, 186, and 107 for (B), and *n* = 307, 118, 135, 105, 117, 122, and 81 for (C,D)). E) Representative images of PPAR‐γ staining in hMSCs before and after moving out of short and long microchannels with widths of 10 and 3 µm on Day 7. PPAR‐γ, cyan; Actin, red; Nucleus, blue. Scale bar: 50 µm. F–H) N/C ratio F), relative nuclear mean intensity G), and relative cellular mean intensity H) of PPAR‐γ in hMSCs (*n* = 360, 297, 275, 200, 203, 125, and 80 for (F), and *n* = 289, 107, 86, 77, 81, 54, and 34 for (G,H). Welch ANOVA with Games–Howell's posthoc test was applied for (B) and (F), while Kruskal–Wallis test with Dunn's multiple comparisons was applied for (C,D) and (G,H). Error bars represent 95% confidence intervals. *p < 0.05, **p < 0.01, ***p < 0.001.

Mechanical cues within tissue microenvironments have been reported to regulate stem cell differentiation.^[^ ^27^ ^]^ To determine if lineage commitment is impacted by confined migration, we used immunofluorescence to visualize the levels and localization of the early osteogenic transcription factors RUNX2 and Osterix.^[^ ^28^ ^]^ Confined migration induced an upregulation of RUNX2 and Osterix levels in both the cytoplasm and nucleus in nearly all conditions, but cells migrating through long tight microchannels saw the highest increases (**Figure** **4**A; and Figure S7B,C, Supporting Information). Most notably, when measuring the ratio of nuclear signal to cytoplasmic signal, only cells that had traversed long 150 µm channels of either 3 or 5 µm widths were found to have significant increases in RUNX2 translocation, with long narrow channels showing an increase of over 40% after 7 days of culture (Figure 4B–D). This response was not observed after 4 days (Figure S8, Supporting Information), similar to other osteogenic differentiation mechanisms.^[^ ^29^ ^]^ Interfering with YAP signaling was found to prevent confinement‐sensitive osteogenic differentiation, as cells treated with the YAP inhibitor verteporfin did not exhibit similar levels of RUNX2 translocation after 7 days (Figure S9, Supporting Information). Confinement did not appear to select cells that were precommitted to an osteogenic fate, as culturing cells in osteogenic media did not result in enhanced confined migration (Figure S10, Supporting Information). To better understand late stage osteogenic differentiation, we assessed alkaline phosphatase (ALP) activity in stem cells after 7 and 14 days of culture before and after confined migration. While an enhancement in ALP activity is observed in cells after permeating narrow 3 µm channels after 7 days (Figure S7A, Supporting Information), this enhancement is dependent upon joint stimulation with both confinement and osteogenic media after 14 days. Stimulation with both osteogenic media and confinement was synergistic, as the highest levels of ALP activity were found in cells exposed to osteogenic media after migration through narrow 3 µm confinement (Figure S11, Supporting Information).

To assess the specificity of confinement‐induced fate specification, we also measured the response of the adipogenic transcription factor PPAR‐γ (Figure 4E–H).^[^ ^30^ ^]^ Similar to RUNX2, all channel patterns induced an upregulation of transcription factor levels in both the nucleus and cytoplasm (Figure 4G,H). Interestingly, and opposite to the RUNX2 pattern, the nuclear translocation ratio of PPAR‐γ fell as channel length increased and channel width decreased, suggesting that confinement works to suppress adipogenic differentiation (Figure 4F). Expression and localization of the chondrogenic transcription factor SOX9 was also examined as a function of confinement length and width. While SOX9 was found predominantly in the nucleus, no significant difference in its expression or localization as a function of confinement was found (Figure S12, Supporting Information).

In our microchannel system (and indeed, in all currently available microchannel systems), a relatively small number of cells exit into a covered reservoir, making it challenging to collect enough cells for downstream analyses requiring thousands of treated cells. To address this, we built a new confinement device dubbed the “TRAP” chip (Trap‐based Recovery After Permeation) (Figure S13, Supporting Information) to collect either cells or lysates of cells postconfinement. By surrounding precisely‐cut microchannel exits with a solid ring of PDMS, we were able to substantially decrease the fluid volume required for trypsinization or lysis in outer reservoirs. Using these TRAP chips, we performed qPCR and Western Blots on lysates taken from day 7 postconfinement cells to validate our previous findings (Figure S14A, Supporting Information). qPCR results showed an upregulation of both YAP and RUNX2 mRNA post‐3 µm confinement (Figure S14B, Supporting Information), while western blots showed a large increase in RUNX2 protein levels post‐3 µm confinement (Figure S14C, Supporting Information). These results, which support our immunofluorescence findings, are the first quantifications of mRNA levels or bulk protein levels in cells following sustained confined migration.

To address whether RUNX2 and/or PPARγ translocation represent “default” pathways for hMSCs in 2D, we plated stem cells on collagen‐coated glass coverslips for 7 days and found that simple sustained culture does not induce osteogenic transcription factor translocation (Figure S15A,B, Supporting Information), further strengthening our finding that early lineage commitment is a function of confinement pattern. In contrast, stem cells grown for 7 days did spontaneously start to display increased PPARγ translocation (Figure S15C,D, Supporting Information), underscoring the inhibitory effect of confinement on adipogenesis.

### 2.5 Confined Migration Drives Histone Acetylation

**Figure 5:**
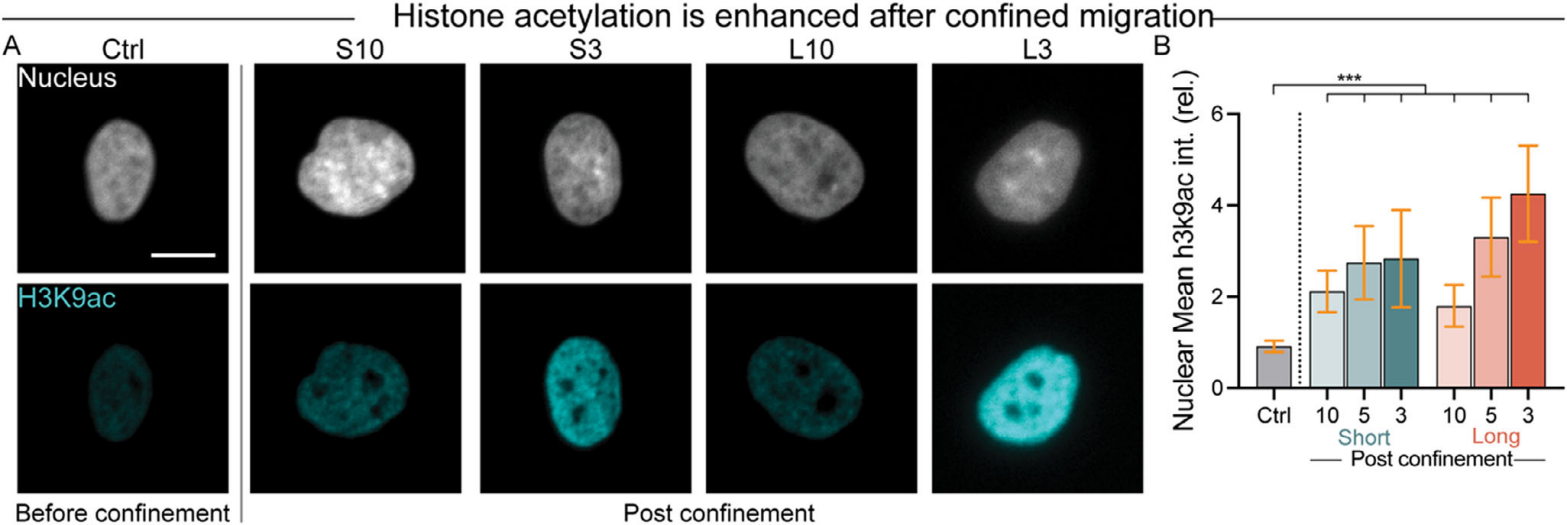
Confined migration drives histone acetylation. A) Representative images of H3K9ac and DNA staining in cells before and after migrating through 10 and 3 µm wide microchannels on Day 7. H3K9ac, cyan; Nucleus, gray. Scale bar: 10 µm. B) Quantification of the relative nuclear mean intensity of H3K9ac in cells before and after migrating through different microchannels on Day 7 (*n* = 389, 138, 66, 100, 99, 81, and 52). Kruskal–Wallis test with Dunn's multiple comparisons was applied for (B). ***p < 0.001. Error bars represent 95% confidence intervals.

As stem cell differentiation is an inherently nuclear process, we turned our attention to confinement‐induced changes in epigenetic organization of the nucleus. It has been documented that nuclear deformation can induce chromatin reorganization and epigenetic remodeling,^[^ ^14^, ^15^, ^16^ ^]^ triggering signaling events that result in altered cell behaviors.^[^ ^31^ ^]^ One of the primary mechanisms governing early cell differentiation involves epigenetic modifications, including histone methylation and histone acetylation.^[^ ^32^, ^33^ ^]^ The acetylation of the 9th residue on histone protein 3 (H3K9ac) decondenses chromatin, facilitating transcriptional activities that occur during differentiation. We assessed the acetylation status of H3K9 to better understand epigenetic remodeling in stem cells before and after confined migration (**Figure** **5**A). Compared to unconfined cells, stem cells migrating through confinement displayed enhanced H3K9ac signal. This occurred in a dose dependent fashion, with cells that had traversed long, narrow channels showing the highest acetylation response (Figure 5B). To investigate the general relationship between YAP activity, histone acetylation, and the nuclear translocation of RUNX2, cells were cultured on glass coverslips in osteogenic differentiation media containing the YAP inhibitor verteporfin for 7 days, followed by staining for H3K9ac and RUNX2. YAP inhibition was found to significantly reduce histone acetylation and the nuclear translocation of RUNX2 (Figure S16, Supporting Information), suggesting that YAP signaling plays a key role in regulating histone acetylation and facilitating osteogenic differentiation even outside of confinement.

### 2.6 Nuclear Deformation, not the LINC Complex, Drives Confinement‐Sensitive Stem Cell Differentiation

**Figure 6:**
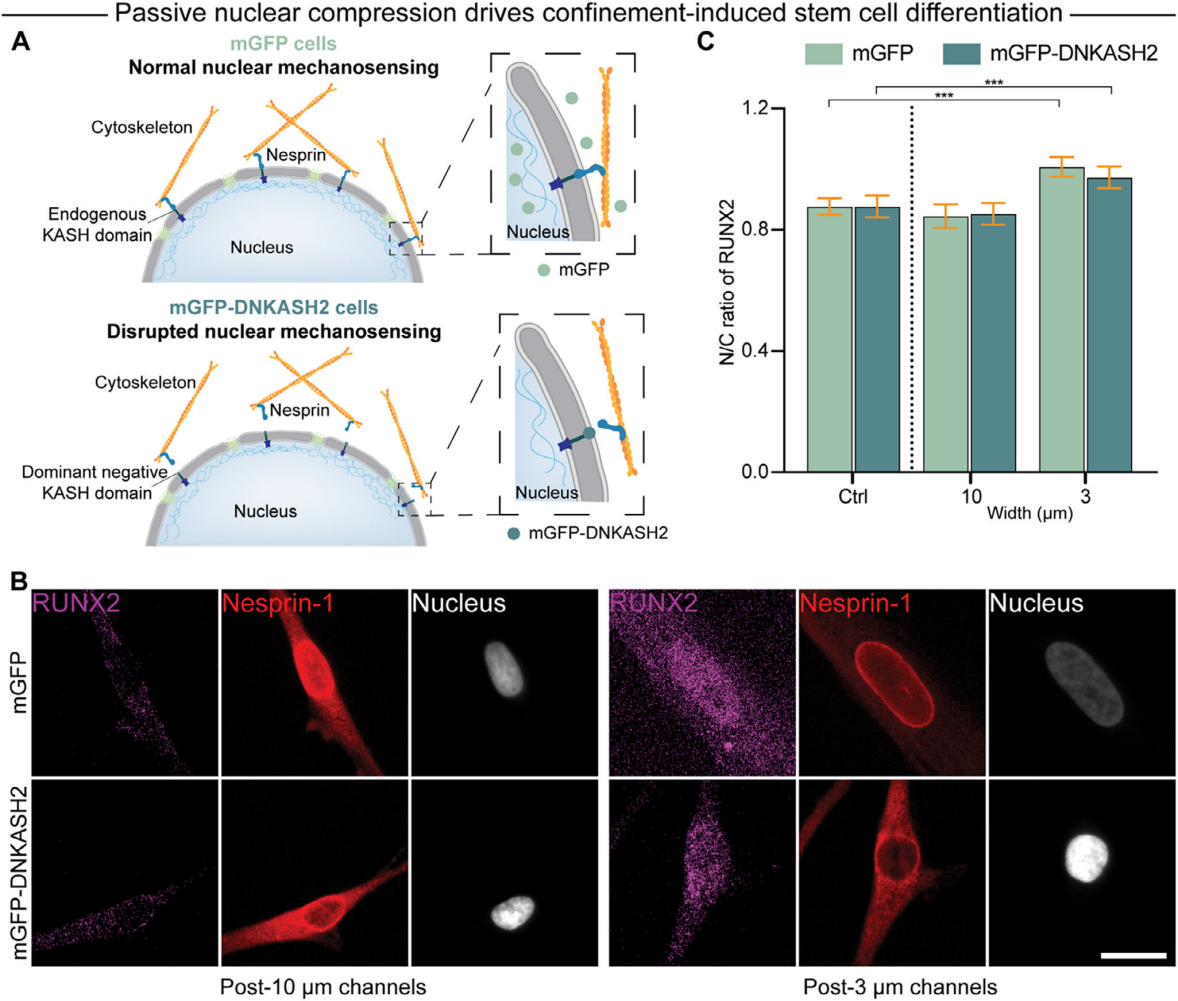
Passive nuclear compression drives confinement‐induced stem cell differentiation. A) Schematic of how the dominant‐negative KASH2 domain fused with mGFP disrupts cytoskeleton‐based nuclear mechanosensing. B) Representative images of RUNX2 staining in mGFP and mGFP‐DNKASH2 hMSCs after migration through long 3 or 10 µm‐wide microchannels on day 7. RUNX2, magenta; Nesprin‐1, red; Nucleus, gray. Scale bar: 20 µm. C) Quantification of N/C ratio of RUNX2 in mGFP and mGFP‐DNKASH2 hMSCs after migration through long 3 or 10 µm‐wide microchannels on day 7 (*n* = 210, 112, 92, 62, 126, and 79). Two‐way ANOVA with Tukey's posthoc comparisons was applied for (C). ***p <  0.001. Error bars represent 95% confidence intervals.

It has become well‐appreciated that mechanical forces can be transmitted from the cell surface to the nucleus via the cytoskeleton.^[^ ^34^, ^35^ ^]^ These forces have been shown to be transmitted across the nuclear envelope through the LINC complex,^[^ ^12^ ^]^ where they ultimately influence chromatin organization and epigenetic remodeling.^[^ ^14^, ^15^, ^16^ ^]^ While previous studies have reported that Nesprin‐2 accumulates at the nuclear front and facilitates nuclear movement through confined spaces,^[^ ^36^ ^]^ we observed homogeneous Nesprin‐1 distribution during microchannel migration (Figure S17, Supporting Information). In contrast with LINC complex‐induced changes in nuclear organization, it has also been shown that direct nuclear compression can also give rise to different cell behaviors independent of the cytoskeleton.^[^ ^21^ ^]^ To determine which regime of nuclear deformation contributes most to confinement‐induced stem cell differentiation, we introduced an mGFP‐tagged dominant‐negative KASH2 (DN‐KASH2) domain nesprin construct into hMSCs to sever the force‐bearing connection between the cytoskeleton and the nucleus (**Figure** **6**A). This resulted in displacement of endogenous nesprin to the cytoplasm which could be visualized in fixed cells stained for Nesprin‐1 (Figure 6B). In long 10 µm channels, both our mGFP control stem cells and the mGFP‐DN‐KASH2 stem cells did not exhibit an increase in RUNX2 nuclear translocation, in agreement with our previous results with wild type (WT) hMSCs. In long 3 µm channels, where WT hMSCs exhibited the highest degree of RUNX2 nuclear translocation, both the mGFP and the mGFP‐DN‐KASH2 hMSCs exhibited similar levels of confinement‐induced RUNX2 translocation (Figure 6B,C), suggesting that confinement‐sensitive stem cell differentiation is driven primarily by simple nuclear deformation, as opposed to cell‐induced traction forces traveling through the LINC complex and altering chromatin state.

Standard lentiviral transduction requires fluorescence‐activated cell sorting and puromycin selection to isolate positively‐transduced cells, which introduces the potential for shear force‐ and chemical‐induced confounding effects. To minimize this, we seeded cells directly in the microchannel chips without any sorting, resulting in a mixed population of labeled cells (transduced) and unlabeled cells (untransduced) within the same population, allowing for direct comparison of cell populations within the same chip (Figure S18A, Supporting Information). Using this approach, we found that untransduced and transduced cells both display similar levels of RUNX2 nuclear translocation (Figure S18B,C, Supporting Information), further strengthening our conclusion that confinement‐induced stem cell differentiation occurs as a function of nuclear deformation.

## 3 Discussion

Human mesenchymal stem cells (hMSCs) have emerged as powerful candidates for many clinical therapies,^[^ ^1^ ^]^ yet their full potential for tissue engineering and cell therapy has not been reached. One major reason for this is the fact that in many applications, the vast majority of injected stem cells do not successfully engraft within the tissue of interest,^[^ ^37^, ^38^, ^39^, ^40^ ^]^ and even when they do, they do not demonstrate proper differentiation.^[^ ^41^, ^42^, ^43^ ^]^ Indeed, some have found that stem cell‐secreted anti‐inflammatory factors and other signaling molecules are likely the primary drivers of the observed therapeutic effects of stem cell therapy.^[^ ^44^, ^45^, ^46^ ^]^ However, engineering approaches have resulted in a greater understanding of the mechanical cues that can drive stem cell differentiation, providing a strong platform from which deeper control of stem cell behavior can be gained. To continue to build on this approach, it is imperative to understand the interactions between hMSCs and the diverse mechanical cues presented in vivo and subsequent consequences, given their migratory nature.^[^ ^47^ ^]^ Stem cells have been shown to migrate through diverse tissues postinjection, meaning that the cues they experience during this migratory journey likely play a major role in their ultimate fate and function.^[^ ^48^, ^49^ ^]^ Here, we show that this self‐induced migratory journey, through levels of confinement found in tissues in vivo, can drive stem cells toward an osteogenic fate. We have also identified the deformation‐based mechanism through which this mechanical confinement influences stem cell behavior, providing an important starting point from which the field can continue to optimize in vitro control of therapeutic cells.

Previous research has shown that immune cells and metastatic cancer cells can undergo a mesenchymal‐to‐amoeboid transition under extreme confinement,^[^ ^24^ ^]^ downregulating their focal adhesions, reorganizing their cytoskeleton, and increasing their migration speed. We have found a similar pattern in stem cells, as they increase their speed when traversing very narrow channels. Interestingly, we did not observe the classic blebbing morphology that occurs in immune cells and cancer cells in tight confinement. Our observations of morphological changes of cells and their nuclei during confinement is in agreement with previous studies,^[^ ^7^ ^]^ and we have gone a step further to quantify the degree to which these changes are retained after exit from confinement, mirroring cellular arrival at sites of healing or inflammation in vivo. The ability of these stem cells to exhibit a mechanical memory of their journey shows an integration of mechanical stimuli on the timescale of days, altering epigenetic state and opening the door for changes in gene expression as a function of confinement. It also provides a new potential frame of reference from which to study other migratory cells that mobilize in response to long range stimuli,^[^ ^50^ ^]^ including those that are recruited to sites of wound healing or inflammation. The future inclusion of microchannel stiffness will also contribute to our understanding of the interplay between mechanosensing and self‐imposed confinement, although recent observations that cells confined in soft microchannels can greatly deform the channel walls^[^ ^51^ ^]^ means that these soft systems would not allow for systematic investigation of the precise relationship between nuclear deformation and consequent cell behavior and fate.

hMSCs have been shown to be highly mechanosensitive across a wide range of physiological stiffnesses.^[^ ^52^ ^]^ Thus, a deeper understanding of their engagement with mechanical cues present in the body, as well as the mechanisms underlying resultant mechanosensitive lineage‐specific differentiation, is crucial for the development of hMSC‐based stem cell therapies.^[^ ^6^, ^53^ ^]^ Previous studies have demonstrated that epigenetic regulation plays a significant role in controlling the differentiation potential of hMSCs.^[^ ^18^ ^]^ We have found that stem cells experience an increase in histone acetylation after sustained migration through long, narrow 3 µm channels, opening up new regions of chromatin and potentiating changes in gene expression. Interestingly, cancer cells have been found to respond to much shorter narrow channels with global increases of heterochromatin in intergenic regions, an enhancement of specific promoter accessibility, and the dynamics of nuclear condensates.^[^ ^54^, ^55^ ^]^ These short, narrow, “pinch‐point” style constrictions have also been found to stimulate the polarization of nesprin at the leading edge of the nucleus.^[^ ^36^ ^]^ As we did not observe a polarization of nesprin in our sustained confining microchannels, it is likely that the modalities through which cells permeate either short or sustained narrow confinements are distinct, most likely due to the inability of cells to establish large protrusions and force‐bearing focal adhesions at the opposite end of a long microchannel. This confinement‐specific pattern of epigenetic modification and nuclear envelope organization suggests that patterns of confinement alone may play a role in the accessibility and activity of different regions of the genome. While microchannels are ideal tools for observing dynamic cellular responses to confinement and probing the expression and localization of specific mechanosensitive proteins,^[^ ^56^ ^]^ one inherent limitation in these systems is an inability to collect large numbers of cells postconfinement for high content molecular analysis. Thus, future work focusing on the development of high‐content devices compatible with modern omics tools will likely reveal a great deal about confinement‐specific gene expression and epigenetic signaling.

In addition to our findings on epigenetic responses to confinement, we have shown here that stem cells maintain a persistent mechanical memory of their migration through physiological confinement, even after exiting into an unconfined space. Most simply, this can be observed in the plastic changes to cell and nuclear morphology, with significant and persistent modifications in nuclear aspect ratio representing the most interesting cellular adaptation, given the key role of the nucleus in mechanosensitive stem cell differentiation.^[^ ^57^ ^]^ This concept of stem cell mechanical memory has been observed in response to sustained culture on 2D substrates with different stiffness cues.^[^ ^58^ ^]^ Similar to our findings, these systems show that substrate mechanics can drive both YAP translocation and RUNX2 signaling for days after dosing. Interestingly, while the effects of 2D substrate stiffness cues were found to quickly wane when cells were transferred to new environments, our observation of persistent and robust RUNX2 signaling after a confinement cue that only lasts hours suggests that confinement may either serve as a more powerful mechanical cue or induce alternative signaling mechanisms within stem cells. Several studies have emphasized the critical role of nuclear deformation in guiding stem cell lineage specification in response to various mechanical stimuli. For example, osteogenesis has been shown to be enhanced in stem cells cultured on convex surfaces.^[^ ^59^ ^]^ As these surfaces result in significant nuclear deformation compared to concave or flat surfaces due to downward force from the actin cap, this is in line with our observations of osteogenesis after nuclear deformation in confinement. Surface microtopography has also been used to induce nuclear deformation in stem cells, with optimal patterns driving osteogenesis both in vitro and on implant surfaces in vivo.^[^ ^23^ ^]^ This deformation was also associated with dynamic changes in chromatin organization,^[^ ^23^ ^]^ similar to our findings in stem cells within fully confining environments. The fact that the deformation of stem cell nuclei can serve as a powerful cue for fate specification is underscored by the high level of nuclear deformability in naïve embryonic stem cells, especially given that nuclear stiffness can increase sixfold during the process of terminal differentiation.^[^ ^60^ ^]^

As differentiation also represents, almost by definition, a plastic, nonreversible change in cell state, our observation of confinement‐sensitive osteogenesis in stem cells further underscores the powerful stimulus that physiological confinement can present. This observation is supported by a small yet compelling body of literature that has examined changes in stem cell fate as a function of self‐directed migration through mechanical constriction; one of the only studies to have investigated this phenomenon utilized transwell assays to show that constricted migration through short and narrow transwell assay membranes enhanced osteogenic media‐induced differentiation in hMSCs as a function of pore diameter, but only when postmigration cells were at a low plating density.^[^ ^8^ ^]^ More recently, it was also shown that mechanically confining cells in “pits” of defined volume could also stimulate osteogenesis,^[^ ^61^ ^]^ further highlighting that 3D confinement can serve as a powerful mechanical cue to modulate stem cell fate. This is further supported by the fact that hyperacetylation of osteogenic promoters occurs on H3K9,^[^ ^62^, ^63^ ^]^ which we have shown here to be significantly upregulated in response to specific levels of sustained confinement. As this H3K9ac regulation can be induced by specific histone acetyltransferases (HATs) in hMSCs,^[^ ^64^ ^]^ and as stem cell nuclei show major changes in cellular and nuclear volume when measured rapidly in confinement,^[^ ^65^ ^]^ future work investigating the connection between nuclear deformation and HATs like KAT2A/Gcn5 and KAT2B/PCAF and their interactions with nuclear envelope^[^ ^66^, ^67^, ^68^ ^]^ will be of great importance. While we primarily measure our differentiation potential in terms of nuclear to cytoplasmic ratio, it is interesting to note that the overall levels of PPAR‐γ were found to increase with confinement, even though the nuclear translocation was lower. This hints that confinement may stimulate an overall increase in the transcription and translation of transcription factors for multiple signaling pathways, but the ultimate translocation of these factors from the cytoplasm into the nucleus for fate specification is more dependent on the specific degree of confinement.

**Figure 7:**
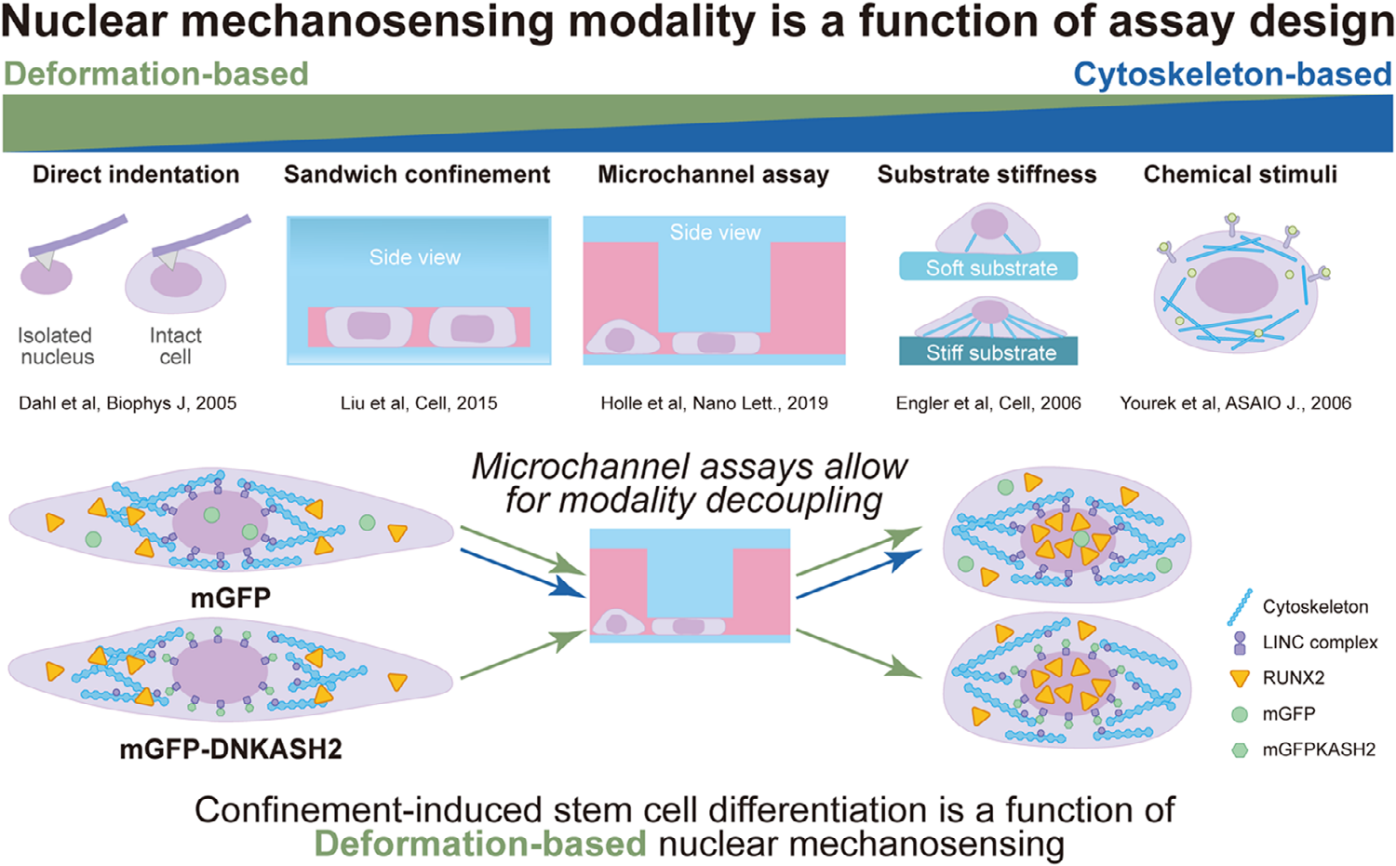
Confinement‐induced stem cell differentiation is a function of deformation‐based nuclear mechanosensing. Assay design plays a major role in the types of nuclear mechanosensing modalities that can be tested. A directly indented nucleus will not reveal the effect of force across the LINC complex, and the inhibition of actomyosin contractility will not give specific answers about nuclear deformation. Most other assays fall somewhere in the middle. By utilizing microchannel assays in conjunction with engineered cells, these two modality mechanisms can be effectively decoupled. In this case, we find that confinement‐induced stem cell differentiation is more dependent on passive nuclear deformation than active force transduction across the nuclear envelope.

In every mechanobiology experiment that results in nuclear morphology change, the possibility for nuclear mechanosensing is present (**Figure** **7**). In nearly all of these experiments,^[^ ^24^, ^69^, ^70^, ^71^, ^72^ ^]^ the nucleus is both deformed and exposed to force across the nuclear envelope. Thus, confinement mechanobiology experiments must grapple with two competing modalities of nuclear mechanotransduction: deformation‐based nuclear mechanosensing and cytoskeleton‐based nuclear mechanosensing. While these two modalities are intricately intertwined, and thus difficult to control for, some well‐designed investigations have managed to decouple their effects. Deformation‐based mechanosensing occurs as a function of bulk nuclear shape change, which has been shown in isolated nuclei, completely disconnected to the cytoskeleton, to result in major changes in chromatin organization.^[^ ^69^ ^]^ Cytoskeleton‐based nuclear mechanosensing occurs as a function of force across the LINC complex, which translates cytoplasmic mechanical signals into changes at lamin‐associated domains of chromatin, as has been observed via magnetic twisting cytometry experiments that tug on single integrins, resulting in changes in gene expression without major changes in nuclear shape.^[^ ^73^ ^]^ In reality, most nuclear mechanotransduction experiments fall somewhere along a gradient of the two (Figure 7); for example, nuclear deformation on a slow scale that allows for cytoskeletal adaptation to the compressive force would likely engage both mechanosensing mechanisms. While these effects are likely synergistic in most cases, it is nonetheless interesting to determine which mechanism contributes more to the mechanosensitive response. For instance, it has been previously reported that cytoskeleton‐based nuclear mechanosensing plays a crucial role in cardiac development by modulating chromatin organization. In mouse cardiomyocytes with a disrupted LINC complex, peripheral H3K9me3‐marked chromatin was lost and the expression level of cardiac genes decreased.^[^ ^74^ ^]^ In contrast, another study demonstrated that in hMSCs with nuclei disconnected from the cytoskeleton, the nuclear response to deformation as measured by lamin A/C organization and chromatin conformation was equivalent to wild type cells, indicating that deformation‐based nuclear mechanosensing may be the predominant mechanism under such conditions.^[^ ^23^ ^]^ Here, we have taken this a step further and determined that confinement‐sensitive stem cell differentiation is primarily due to deformation‐based nuclear mechanosensing, as populations of stem cells that have had their nuclei disconnected from the cytoskeleton show similar levels of confinement‐induced differentiation as wild type cells. Unraveling this interplay is not just an exercise in curiosity‐by understanding which mechanism plays a more prominent role, the downstream modulators of this process can be directly perturbed in the future, giving more control over cell fate for translational cellular engineering applications.

Our observations on stem cell differentiation in confinement and our findings on the nuclear mechanism of this phenomenon also provide important insight into other observations of mechanosensitive stem cell differentiation in diverse 3D biomaterials. For example, it has been shown that fibronectin‐ and gelatin‐coated silk fibroin scaffolds with an average pore size of 173.8 µm are more effective at inducing osteogenesis than scaffolds with larger pore sizes.^[^ ^75^ ^]^ In another study, stem cells seeded on low porosity bioscaffolds made from nonwoven fabrics showed higher alkaline phosphatase activity and osteocalcin expression than those with higher porosity.^[^ ^76^ ^]^ As volume regulation has been shown to drive osteogenic differentiation in conflicting and context‐specific fashions, the degree to which 3D biomaterial mechanical properties can dictate stem cell volume is an important question when attempting to regulate fate.^[^ ^77^, ^78^ ^]^ Our findings also offer important context to observations made in biomaterials used in vivo for bone repair, given observations that hMSC transplantation in void‐forming hydrogels that impose physiological confinement are more effective at inducing bone regeneration than bulk hydrogels.^[^ ^79^ ^]^ It is important to note that even with pore sizes and void spaces having diameters in the hundreds of micrometers, cell migration in between these interconnected pores or voids exposes cells to much tighter levels of confinement.^[^ ^80^ ^]^ While a number of other confounding factors, including nutrient diffusion, structural stability, mechanical properties, and degradation may all play a role in these biomaterials, here we have isolated the role of confinement and subsequent nuclear deformation, thus providing a roadmap for the development and deployment of novel biomaterials in the future.

Beyond demonstrating that confined migration can directly drive stem cell fate, we hope that this work highlights the need for future investigations focused on directly quantifying the morphologies of interstitial spaces through which stem and progenitor cells migrate in vivo during development, homeostasis, and wound healing. Advances in tissue clearing, expansion microscopy, and intravital imaging are facilitating new findings in this direction, all of which can contribute directly to the engineering of biomaterials and microfluidic systems that can impose precise levels of confinement matching the physiological microenvironment. Given the wide variety of cell behaviors and states that the field of tissue engineering will demand in the near future, these systems can rise to that challenge, providing new insights into cellular mechanobiology and more effective tools for translational cellular engineering.

## 4 Conclusion

Here, we have developed a microchannel‐based system presenting varying degrees of confinement to migratory stem cells in order to investigate the impact of confined migration on differentiation. We demonstrate that after self‐induced exposure to physiological confinement, stem cells show a degree of mechanical memory in the form of sustained alterations in cell and nuclear morphology. Beyond these changes in shape, we find that confinement‐primed stem cells differentiate preferentially down an osteogenic pathway. We also show that this mechanosensitive change in fate is a function of deformation‐based nuclear mechanosensing by ruling out the LINC complex as a primary driver of these outcomes. Ultimately, we believe that these results open up new avenues of investigation in fundamental stem cell mechanobiology and provide a new platform from which the control of stem cell fate can be strengthened.

## 5 Experimental Section

### Cell Culture

Human bone marrow‐derived mesenchymal stem cells were purchased from ATCC (PCS‐500‐012). Cells were expanded in T‐75 tissue culture flasks and maintained in Dulbecco's Modified Eagle Medium (DMEM) (GIBCO,10569‐010) supplemented with 10% fetal bovine serum (FBS) (GIBCO, 26140079) and 1% penicillin/streptomycin (Pen/Strep) (GIBCO, 15140‐122). Cells were cultured at 37 °C in a humidified incubator containing 5% CO_2_ and passaged at 70%–80% confluence. Primary stem cells were not used beyond passage 8.

### Microchannel Chip Fabrication, Assembly, and Cell Seeding

Two‐step photolithography was performed by the Mechanobiology Institute microfabrication core to produce microchannel master molds similar to previous work.^[^ ^65^, ^81^ ^]^ Briefly, a 10 µm thick layer (defining microchannel height) of SU‐8 3010 photoresist (Kayaku, Westborough, MA) was spincoated onto a silicon wafer (BondaTek, Singapore) and crosslinked with UV light via a SUSS Microtec MJB4 mask aligner through a custom sodalime optical photomask based on an Autocad file (defining channel width and length) printed via a MLA 150 maskless aligner (Heidelberg Instruments Mikrotechnik GmbH, Heidelberg, Germany). This was repeated with SU‐8 3050 (Kayaku, Westborough, MA) to form a 100 µm thick layer (defining reservoir height). Noncrosslinked photoresist was developed with SU‐8 developer (Kayaku, Westborough, MA). SYLGARD 184 Polydimethylsiloxane (PDMS) (Dow Corning, Wiesbaden, Germany) was mixed at a 10:1 base to curing agent ratio followed by vacuum degassing for 5 min to remove bubbles. This mixture was poured onto the microchannel master mold and baked for 2 h at 80 °C. The PDMS was then peeled from the mold and cut into square chips containing open entry and outer reservoirs. For TRAP chips (Figure S13, Supporting Information), microchannels were cut precisely at the channel exits to create an open outer reservoir. These chips, together with glass coverslips, were then treated with oxygen plasma (0.7 mbar, 300 W) for 5 min before being carefully placed in contact with each other and moved to an 80 °C oven for 10 min to improve bonding strength. To reduce outer reservoir fluid volume, a square‐shaped PDMS ring was fabricated and attached surrounding the inner chip to “trap” cells after confinement. 100 µg mL^−1^ rat tail type I collagen (Gibco, Carlsbad, CA) was added to the chips, which were then degassed for 5 min before storing overnight at 37 °C. The following day, chips were rinsed in phosphate‐buffered saline (PBS) three times before storing at 4 °C until use.

For cell seeding, collagen‐coated microchannel chips were removed from 4 °C storage, rinsed in PBS, and placed in a six well plate or glued to the bottom of a well in a six well plate with Picodent dental glue (Picodent, Wipperfürth, Germany) for live imaging before sterilization in UV flight for 30 min. The PBS was then replaced with cell culture media and chips were placed in an incubator for 30 min to equilibrate temperature. Cells of interest were trypsinized and resuspended to a concentration of 1 000 000 or 300 000 cells mL^−1^, depending on the experiment. cell suspension (5 µL) was added to the corners of the inner reservoir. Following this, sufficient cell culture media was carefully added to fill the reservoirs. After 4 h, during which time cells became fully adherent, the chips were fully covered in cell culture media to prevent any gradients in hydrostatic pressure between the inner and outer reservoirs, and to allow for facile diffusion of media. No chemotactic gradients were used for any experiments. For long‐term experiments, media was changed every 3 days. Verteporfin (5 µm, No. 100‐0262, STEMCELL Technologies, Vancouver, Canada) dissolved in dimethyl sulfoxide (DMSO; D8418, Sigma‐Aldrich, Burlington, MA) was used for YAP inhibition experiments. For media‐based differentiation experiments, hMSCs were seeded at a density of 1000 cells cm^−^^2^ on collagen‐coated coverslips and cultured in osteogenic differentiation media (No. 05465, STEMCELL Technologies, Vancouver, Canada) for 7 days.

### Immunofluorescence Staining

Cells on coverslips were fixed in warm 3.7% paraformaldehyde for 15 min at room temperature (RT), while cells in chips were fixed in warm 3.7% paraformaldehyde for 45 min in a shaker at 37 °C. All cells were then rinsed with PBS and permeabilized in 0.2% Triton‐X 100 for 15 min at RT. After three PBS rinses, cells were blocked in 1% bovine serum albumin (dissolved in PBS containing 0.1% Tween‐20) at RT for 30 min, followed by incubation with primary antibodies against H3K9ac (1:200, Abcam, ab4441), RUNX2 (1:100, Abcam, ab76956), PPAR‐γ (1:200, Abcam, ab209350), Osterix (1:200, Thermo Fisher Scientific, BS‐1110R), γH2AX (1:50, Abcam, 195188) Nesprin‐1(1:200, Thermo Fisher Scientific, PA5‐115640), Paxillin (1:200, Abcam, ab32084), and SOX9 (1:200, Abcam, ab185230) at 4 °C overnight. After three PBS washes, samples were incubated with CF568 Phalloidin (1:100, Biotium, 00044) and the following secondary antibodies at RT for 1 h: goat antirabbit CF640 (1:1000, Biotium, 20176), goat antimouse CF640 (1:1000, Biotium, 20175), or goat antirabbit CF488 (1:1000, Biotium, 20019). Counterstaining with Hoechst 33342 (1:5000, Invitrogen, H3570) was performed for 10 min at RT, followed by a triple rinse in PBS. All samples were imaged immediately after the staining.

### Plasmid Production and Lentiviral Transduction

The mGFP‐DNKASH2 plasmid was a gift from Brian Burke's group and was processed by the High throughput Molecular Genomics (HMG) core at the Mechanobiology Institute to insert a lentiviral backbone and produce mGFP plasmid. All plasmids were sequenced and verified by Axil Scientific (Singapore). Lentiviruses were then generated using a Lenti‐XTM Packaging Kit (Takara Bio, San Jose, CA). hMSCs were maintained at 70% confluence in antibiotic‐free media prior to transfection. Viral supernatant was diluted in antibiotic‐free media containing 8 µg mL^−1^ Polybrene (Sigma‐Aldrich, St. Louis, MO, TR‐1003) at a ratio of 1:1 for 24 h. The media was replenished every 24 h. After 72 h, the cells were collected for passaging and seeding.

### Characterization of Cell Migration, Cell Morphology, and Transcription Factor Expression

Live tracking of cell migration was performed using a Celldiscover 7 microscope (Carl Zeiss, Oberkuchen, Germany) with a 20X objective (PIan‐APOCHROMAT 20x/0.7 autocorr Objective). Images were taken every 10 min for 48 h after cell attachment. If a cell started at the entrance of the microchannel and successfully exited the opposite side, it was counted as one migration event. For each image position, the number of migration events and the number of microchannels were recorded. The permeation rate (number of migration events per channel per day) was defined as

(1) $$\mathrm{Permeation}\;\mathrm{rate}\;=\frac{\mathrm{Number}\;\mathrm{of}\;\mathrm{migration}\;\mathrm{events}}{\mathrm{Number}\;\mathrm{of}\;\mathrm{microchannels}\;*\;\mathrm{Number}\;\mathrm{of}\;\mathrm{days}}\;$$

For cells that managed to completely migrate through a microchannel, the center of mass of each cell was manually tracked with the built‐in Manual Tracking plugin in ImageJ (NIH). Tracking started when the center of mass of a given cell entered the microchannel and stopped once it exited the microchannel. Cells that died, returned, or divided during migration were not considered for migration speed analysis. The average migration speed was then calculated in Microsoft Excel.

Immunostained samples were also imaged using a Celldiscover 7 microscope with a 20X objective or an Olympus IX81 Microscope (Olympus, Tokyo, Japan) with a 40X objective (LUCPLFLN‐20X). Images were processed via CellProfiler to obtain cellular area and cellular aspect ratio (assessed via actin staining) and nuclear area and nuclear aspect ratio (assessed via Hoechst staining). Nuclear strain was quantified by measuring the average lengths of the longitudinal and transverse axes before and after confined migration of each biological replicate. Cellular intensity (which is comprised of both cytoplasmic and nuclear signal) and nuclear intensity of transcription factors and histone modifiers were calculated based on the masks generated during area quantification. As the majority of transcription factors are located in or around the nucleus, the tertiary objects module in CellProfiler were employed to define a circular region expanding from the nucleus. The mean intensity in this perinuclear region was used to represent the cytoplasmic mean intensity. Therefore, the nuclear‐to‐cytoplasmic (N/C) ratio of transcription factor was determined as follows

(2) $$N/C\;\mathrm{ratio}=\frac{\mathrm{Mea}n_{n}}{\mathrm{Mea}n_{p}}\;$$

where the Mean_*p*_ and *Mean_n_* are the perinuclear and nuclear mean intensity, respectively. All mean intensities were offset by the background intensity.

To assess focal adhesions, immunostained samples were imaged using a Celldiscover 7 microscope equipped with a 50X water immersion objective (Plan‐APOCHROMAT 50x/0.7 autocorr Objective). Quantification of focal adhesion area and number was performed using CellProfiler. The anisotropy of actin fibers was measured using the previously‐developed ImageJ plugin FibrilTool.^[^ ^82^ ^]^

To measure nuclear volume and height, Z stacks (0.4 µm per slice) of immunostained cells were visualized using an Olympus FV 3000 confocal microscope using a 60X objective (Olympus UPLXAPO 60X Oil Immersion Objective) Images were then analyzed in Imaris 9.7.3 software (Oxford Instruments, Oxford, England) using the surface function. Thresholding was automatically determined by the software while smoothing factor was optimized to ensure accurate rendering of the nucleus. The BoundingBoxOO Length A was defined as the height of the nucleus.

Nesprin positioning was characterized using a previously established method.^[^ ^83^ ^]^ Briefly, a 4‐pixel‐wide line was drawn clockwise along the nuclear periphery, starting from the leading edge of the nucleus relative to the direction of migration. The fluorescence intensity profile was then generated using ImageJ and exported to Excel for further analysis. The profile was divided into four segments: front (first and last 5% of the profile), right (5%–45%), back (45%–55%), and left (55%–95%). The mean intensity of nesprin was quantified for each segment. The intensity values at the front and back were normalized by dividing them by the average intensity of the side segments (mean of the left and right segments).

### Cell Lysate Extraction

Protein lysates or RNA were extracted on day 7 following the established experimental protocol (Figure S14A, Supporting Information). After media removal, chips were washed in cold PBS which was then aspirated from the outer reservoirs while retaining PBS in the inner reservoir. These chips were then placed in a −80 °C freezer. Once the PBS in the inner reservoir was frozen, 100 e of RIPA lysis buffer (89 900, Thermo Fisher Scientific, Waltham, MA) supplemented with a protease and phosphatase inhibitor cocktail (1861281, Thermo Fisher Scientific, Waltham, MA) was added to the outer reservoir only. The outer reservoir was then rigorously scraped with a pipette tip and the resulting lysate was transferred to another trap chip to ensure that all extracted proteins were pooled into a minimal volume of lysis buffer. A total of six chips were used for protein extraction for each microchannel width. For RNA extraction, a similar protocol was utilized but with 100 µLy of Trizol reagent (79 306, Qiagen, Hilden, Germany) instead of RIPA buffer. Protein lysates and RNA extracts were subsequently stored at −80 °C for downstream analyses.

### Western Blot

Protein concentration was quantified using the Pierce BCA Protein Assay Kit (A65453, Thermo Fisher Scientific, Waltham, MA) and adjusted to ensure uniform protein loading across all lanes. Following total protein quantification, equal amounts of protein were mixed with 4× Laemmli buffer containing β‐mercaptoethanol (No. 1610747, Bio‐Rad, Hercules, CA) and denatured for 5 min at 95 °C. Proteins were then separated on a 10% polyacrylamide gel at 100 V for 90 min, then transferred to an Immun‐Blot Low Fluorescence PVDF Membrane (Bio‐Rad, Hercules, CA) using the Trans‐Blot Turbo Transfer System (Bio‐Rad, Hercules, CA) according to the manufacturer's protocol. Following transfer, the membrane was washed with Tris‐buffered saline (20 mm Tris, pH 7.6; 150 mm NaCl) containing 0.05% Tween‐20 (TBS‐T), then blocked with 5% BSA in TBS‐T for 1 h at room temperature. The membrane was incubated with primary antibodies against RUNX2 (1:1000, Abcam, ab76956) and glyceraldehyde‐3‐phosphate dehydrogenase (GAPDH, 1:1000, PA1‐987, Thermo Fisher Scientific, Waltham, MA) at 4 °C overnight on a shaker. After incubation, the membrane was washed three times with TBS‐T (5 min per wash) and incubated with either goat antirabbit secondary antibody (31460, Thermo Fisher Scientific, Waltham, MA) or goat antimouse secondary antibody (31430, Thermo Fisher Scientific, Waltham, MA) at a 1:10000 dilution for 1 h at room temperature. Three additional 5‐min washes with TBS‐T were performed before chemiluminescent detection using a Clarity Western ECL Substrate (1705060, Bio‐Rad, Hercules, CA) for 5 min at room temperature. Imaging was conducted using a ChemiDoc Touch Imaging System (iBright FL1000, Thermo Fisher Scientific, Waltham, MA). Protein expression levels were normalized to the housekeeping protein GAPDH, and signal intensity was quantified using ImageJ software (National Institutes of Health, USA).

### RT‐qPCR

RNA extraction was performed using the TRIZOL reagent and the RNeasy Micro Kit (74004, Qiagen, Hilden, Germany) in accordance with the manufacturer's protocol. Complementary DNA (cDNA) was synthesized from the extracted RNA using the iScript cDNA Synthesis Kit (No. 1708891, Bio‐Rad, Hercules, CA) following the manufacturer's instructions. Quantitative real‐time PCR (qRT‐PCR) was conducted using a CFX96 Touch Real‐Time PCR detection System (Bio‐Rad, Hercules, CA) with SsoAdvanced Universal SYBR Green Supermix (No. 1 725 270, Bio‐Rad, Hercules, CA) in PCR tubes (No. TCS0803, Bio‐Rad, Hercules, CA). The amplification protocol consisted of an initial denaturation step at 95 °C for 30 s, followed by 40 cycles of 95 °C for 5 s and 60 °C for 5 s. A melt curve analysis was performed with 80 cycles, starting at 55 °C and increasing by 0.5 °C every 2 s up to 95 °C. The expression levels of RUNX2, PPAR‐γ, and YAP were quantified and normalized to the housekeeping gene GAPDH using the delta–delta Ct method.

RUNX2 forward sequence 5’‐CGCCTCACAAACAACCACAG‐3’
reverse sequence 5’‐ACTGCTTGCAGCCTTAAATGAC‐3’
PPAR‐γ forward sequence 5’‐ CGAGAAGGAGAAGCTGTTGG‐3’
reverse sequence 5’‐ CTTTGGTCAGCGGGAAGG‐3’
YAP forward sequence 5’‐ CTGAACAGTGTGGATGAGATGG‐3’
reverse sequence 5’‐GAATGGCTTCAAGGTAGTCTGG‐3’
GAPDH forward sequence 5’‐ ACCACAGTCCATGCCATCAC‐3’
reverse sequence 5’‐ TCCACCACCCTGTTGCTGTA‐3’

### Alkaline Phosphatase (ALP) Staining

Cells cultured in chips were maintained under three different conditions: 1) expansion media for seven days, 2) expansion media for 14 days, or 3) expansion media for 7 days followed by osteogenic differentiation media for an additional seven days. Following the respective culture periods, cells were fixed and subjected to ALP staining using the AP Staining Kit (AP100B‐1, System Biosciences, Palo Alto, CA) according to the manufacturer's instructions. Stained samples were imaged using a Mateo FL digital microscope (Leica, Wetzlar, Germany), and image analysis was performed using ImageJ software (National Institutes of Health, USA).

### Statistical Analysis

All data were reported as mean ± 95% confidence interval and analyzed in GraphPad Prism (9.0). Different forms of ANOVA were used for all data with more than two comparisons. One‐way ANOVA (for normally‐distributed data with equal variance) with Bonferroni's posthoc test (for groups with a high variance in sample size), Welch's ANOVA (for normally‐distributed data with unequal variance) with Games–Howell's posthoc test (when sample size is greater than 50) or Dunnett's T3 posthoc test (when sample size is less than 50), and the Kruskal–Wallis test with Dunn's multiple comparisons (for non‐normally‐distributed data with heteroscedastic distributions) were used when appropriate. Two‐way ANOVA with Tukey's posthoc test was used for experiments with two independent variables, normally‐distributed data, and low variance in sample size. *P*‐values less than 0.05 were considered statistically significant.

## Conflict of Interest

The authors declare no conflict of interest.

## Author Contributions

A.W.H. and X.G. conceptualized and designed the study and wrote the manuscript. X.G., Y.L., N.J.W.L., J.Z., and V.R. performed experiments. X.G. analyzed and visualized the data. J.M. drew the schematics. A.W.H. supervised the study.

## Supporting information



Supporting Information

## Data Availability

The data that support the findings of this study are available from the corresponding author upon reasonable request.
