# Supplementary material for: Confined Migration Drives Stem Cell Differentiation
Source: Adv Sci (Weinh). 2025 May 8;12(21):2415407. doi: 10.1002/advs.202415407 (PMC12140319; doi:10.1002/advs.202415407)
Supplement: Supplementary file 1 — Supporting Information [file ADVS-12-2415407-s001.pdf]

ADVANCED SCIENCE
Open Access

Supporting Information

for *Adv. Sci.*, DOI 10.1002/advs.202415407

Confined Migration Drives Stem Cell Differentiation

*Xu Gao, Yixuan Li, Jia Wen Nicole Lee, Jianxuan Zhou, Vaishnavi Rangaraj, Jennifer Marlena and Andrew W. Holle**

Supporting Information

Confined migration drives stem cell differentiation

*Xu Gao, Yixuan Li, Jia Wen Nicole Lee, Jianxuan Zhou, Vaishnavi Rangaraj, Jennifer Marlena, and Andrew W. Holle**

This PDF file includes:

Table S1.
Figures S1 to S18.

Table S1. Measurements of major axis and minor axis of the nucleus before and after migrating through long microchannels with different widths.

Group	Major axis (μm)	Longitudinal strain (%)	Minor axis (μm)	Transverse strain (%)
Ctrl	19.55 ± 0.60	N.A	14.25 ± 0.07	N.A
L10	19.88 ± 0.70	1.70 ± 1.97	13.48 ± 0.22	-5.38 ± 1.10
L3	19.23 ± 0.90	-1.63 ± 1.22	11.88 ± 0.03	-16.55 ± 0.50

*Data was presented as mean ± SD. N.A Not available.

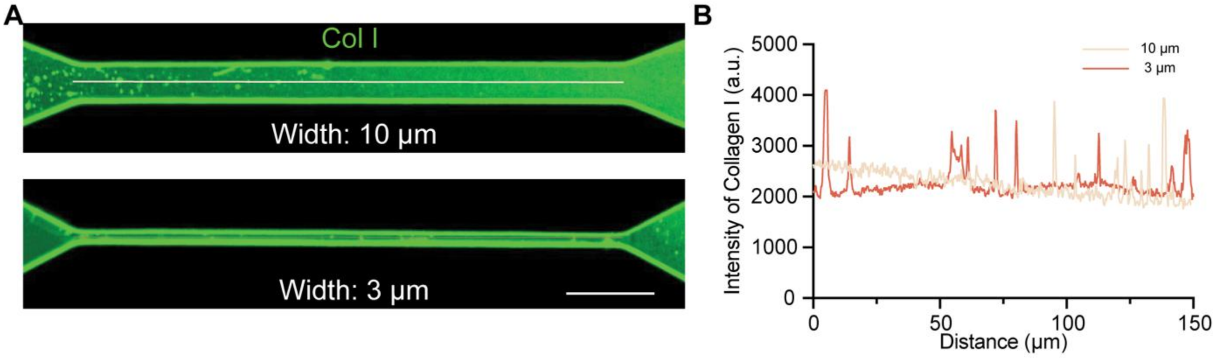

Figure S1. Collagen coating in 10 µm-wide and 3 µm-wide long microchannels. A) Representative collagen I staining in 10 µm-wide and 3 µm-wide long microchannels. Collagen I, green. Scale bar: 25 µm. B) Fluorescent intensity profiles of lines drawn in A. Light red line represents 10 µm-wide long microchannels and deep red line represents 3 µm-wide long microchannels.

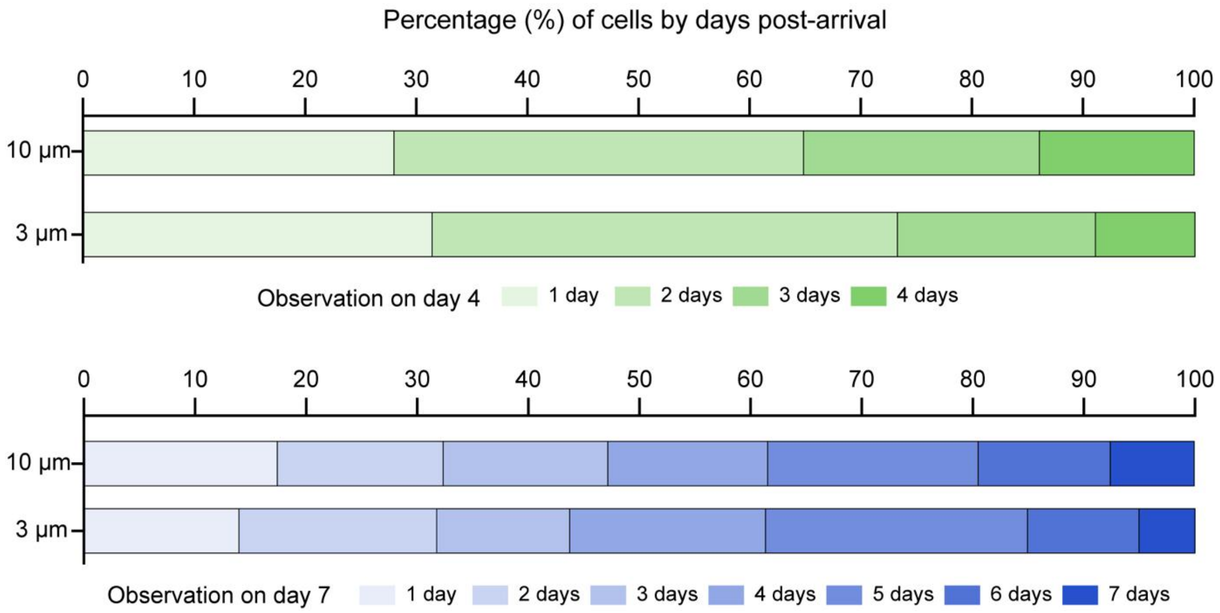

Figure S2. Estimation of the percentage of cells by days post-arrival for 150 µm-long microchannels.

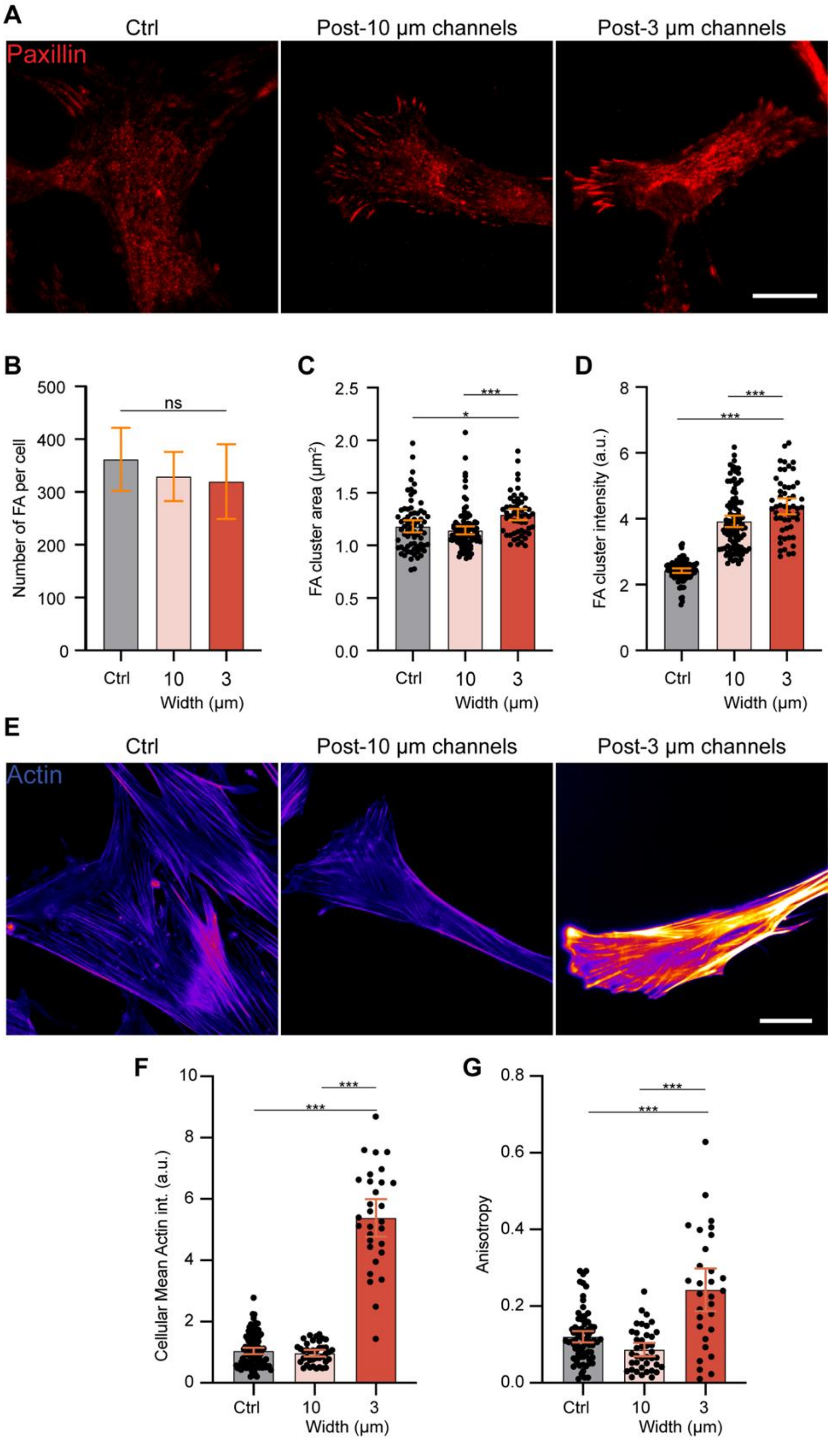

Figure S3. Confined migration induces formation of mature focal adhesions and actin. A) Representative images of paxillin staining in cells before and after exiting long microchannels (150 μm) with widths of 10 μm and 3 μm on Day 7. Scale: 25 μm. B-D) Quantification of the number (B), cluster area (C), and cluster intensity (arbitrary units). (D) of focal adhesions. (n = 87, 103, and 53). E) Representative staining images of actin staining in cells before and after moving out of long microchannels (150 μm) with widths of 10 μm and 3 μm on Day 7. Scale: 25 μm. F-G) Quantification of the cellular actin intensity (arbitrary units) and anisotropy of actin fiber arrays. (n = 76, 40, and 30). One-way ANOVA with Bonferroni's post-hoc test was applied for B. Brown-Forsythe and Welch ANOVA with Games-Howell's

post-hoc test was applied for C, D, F, and G. *P< 0.05.***P< 0.001. ns, no significant difference.

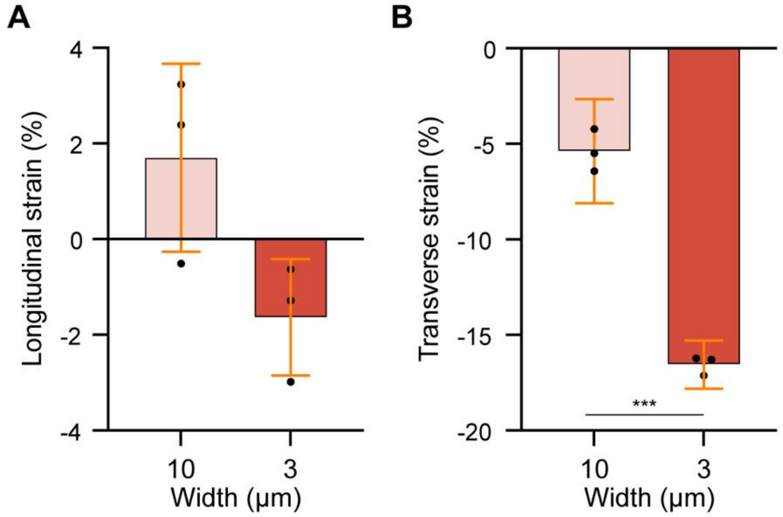

Figure S4. Anisotropic nuclear deformation after confined migration. A-B) Measurements of longitudinal strain and transverse strain of nuclei after migration through long microchannels with different widths (μm) on day 7 (n = 3). Two-tailed unpaired t tests were applied for both A and B. Data presented as mean ± SD. ***P<0.001.

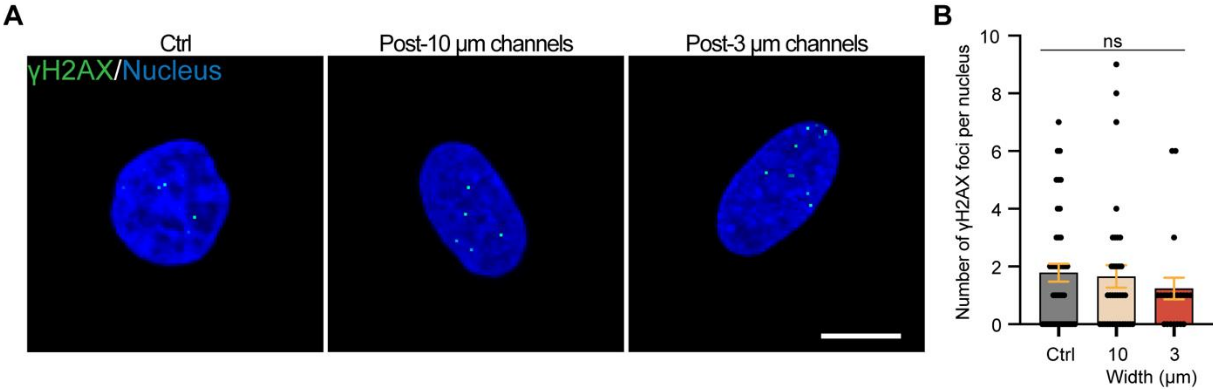

Figure S5. Migration through long narrow microchannels does not cause significant DNA damage. A) Representative images of γH2AX foci in nucleus before and after migration through long microchannels of different widths (μm) on day 7. γH2AX, green. Nucleus, blue. Scale: 10 μm. B) Quantification of the number of γH2AX foci in the nucleus. (n = 42, 35, and 21). Kruskal-Wallis test with Dunn's multiple comparisons test was applied for B. ns, no significance.

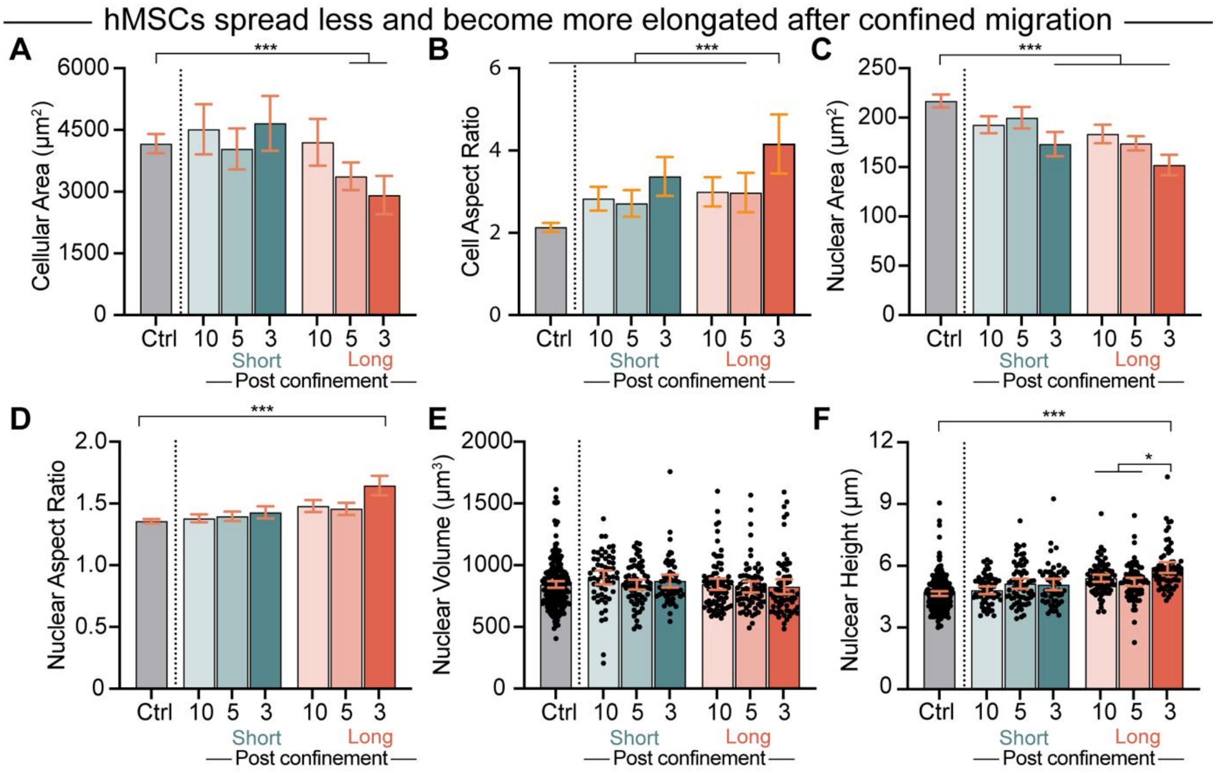

Figure S6. **Cells remained an altered morphological pattern post-confinement on day 7.** A-B) Cellular area A) and cell aspect ratio B) of cells before and after traversing short and long microchannels with different widths. C-D) Nuclear area C) and nuclear aspect ratio D) of cells before and after traversing short and long microchannels with different widths. (n = 269, 146, 109, 90, 105, 106, and 83 for A-B. n = 414, 102, 117, 91, 105, 106, and 83 for C-D.). E-F) Nuclear volume E) and nuclear height F) of cells before and after traversing short and long microchannels with different widths. (n = 212, 58, 67, 54, 77, 71, and 66). Brown-Forsythe and Welch ANOVA with Games-Howell's post-hoc test was applied for A-D. One-way ANOVA with Bonferroni's post-hoc test was used for E-F**.** Error bars represent 95% confidence intervals. *** P< 0.001. ns, no significant difference.

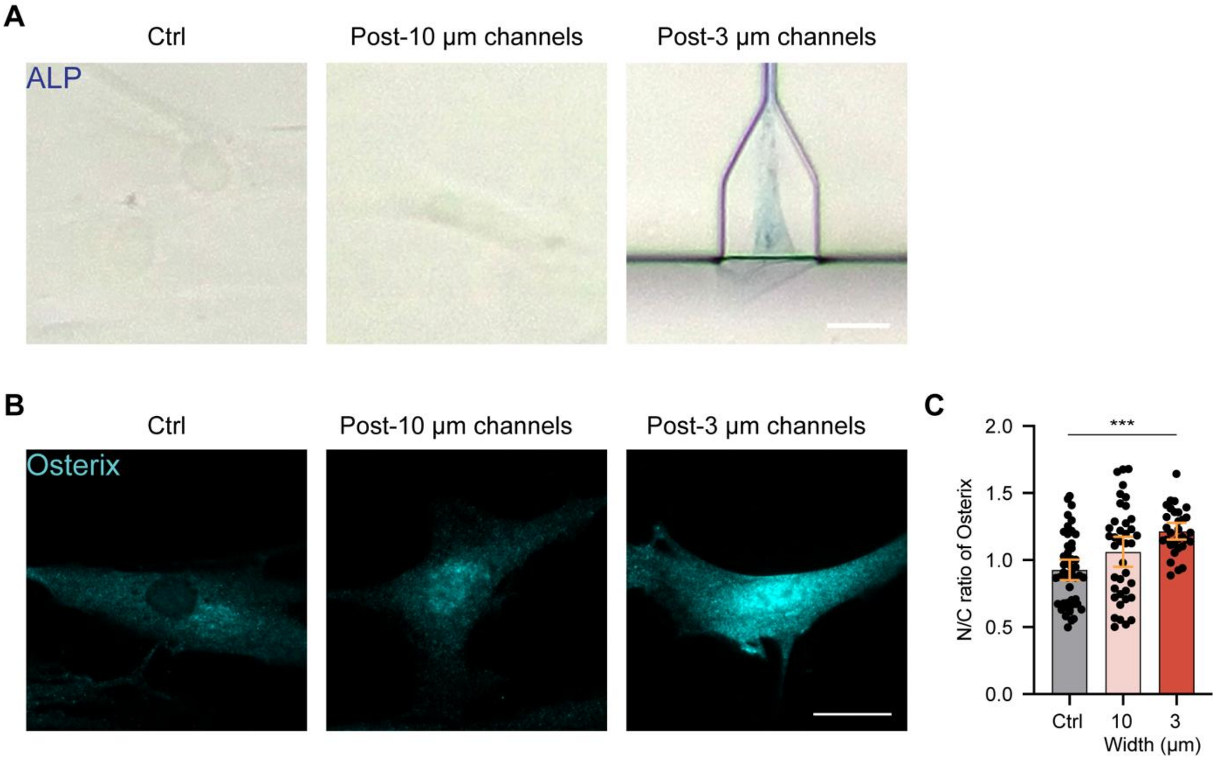

Figure S7. Confined migration drives early osteogenesis. A-B) Representative staining images of ALP (A) and Osterix (B) in hMSCs before and after moving out of long microchannels (150 μm) with widths of 10 μm and 3 μm on Day 7. Osterix cyan. Scale bar: 25 μm. C) Quantification of the N/C ratio of Osterix in cells before and after migrating through different microchannels on Day 7. (n = 48, 39, and 31). Brown-Forsythe and Welch ANOVA with Games-Howell's post-hoc test was applied for C. ***$P< 0.001$.

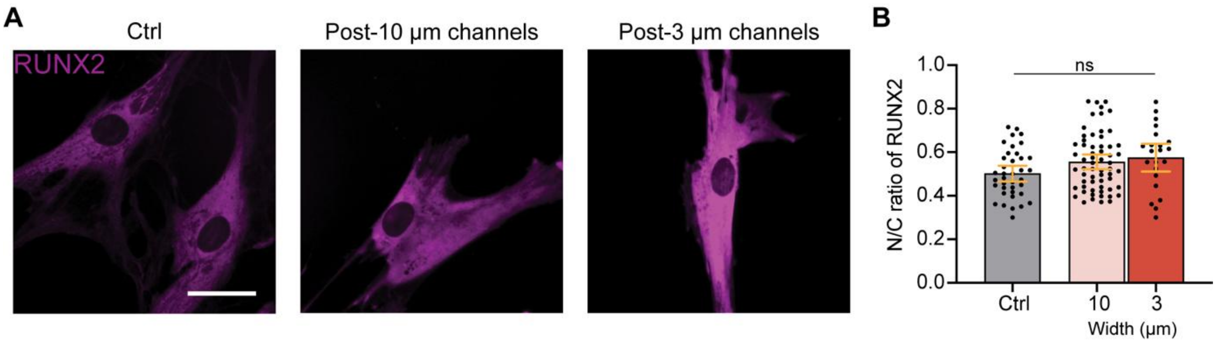

Figure S8. RUNX2 shows no nuclear translocation on Day 4. A) Representative images of hMSCs before and after moving out of long microchannels (150 μm) with widths of 10 μm and 3 μm on Day 4. RUNX2, magenta; Scale bar: 50 μm. B) Quantification of the N/C ratio of RUNX2 in cells before and after migrating through different microchannels on Day 4. (n = 38, 61, and 22). One-way ANOVA with Bonferroni's post-hoc test was applied for B. ns, no significant difference.

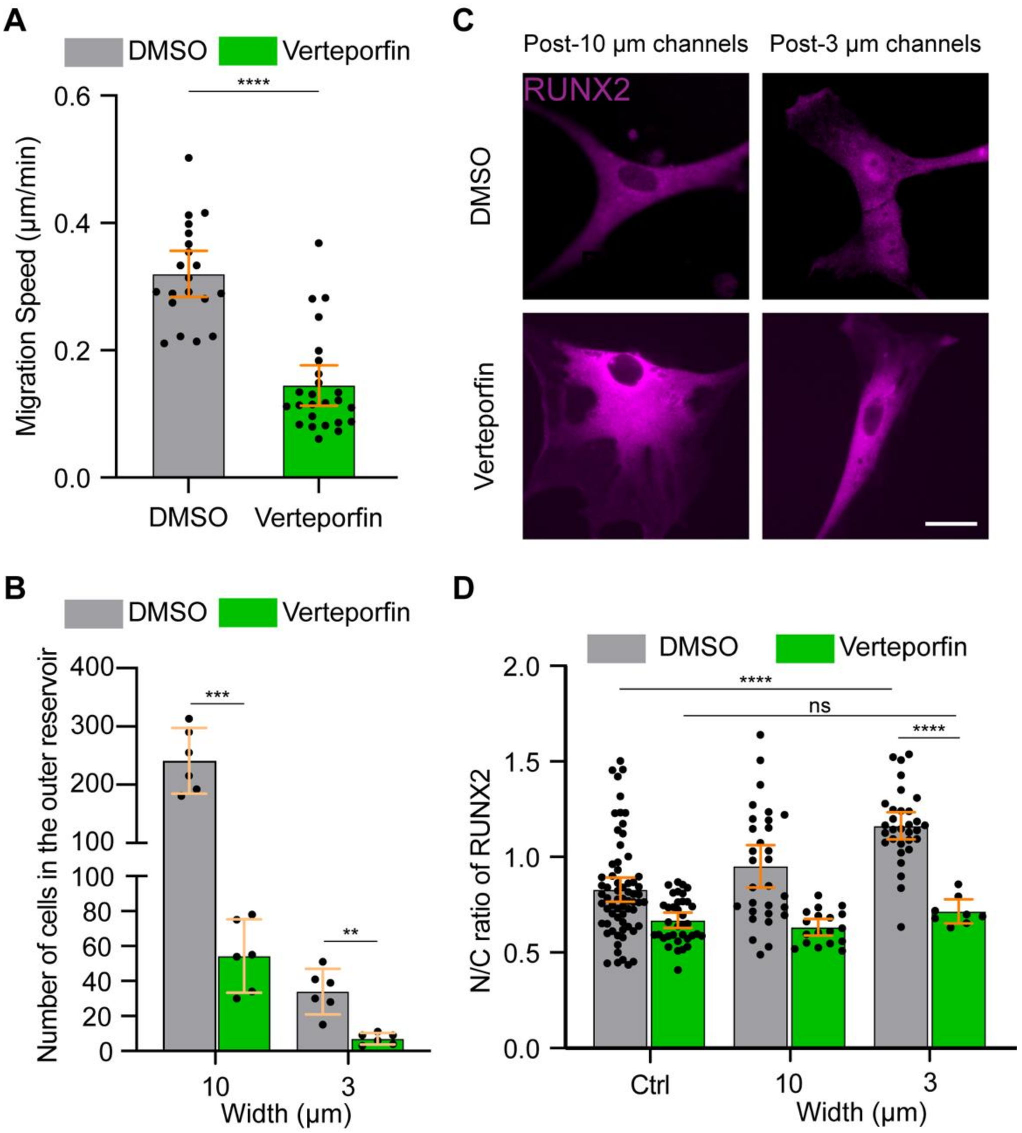

Figure S9. Inhibiting YAP reverse confinement-induced nuclear translocation of RUNX2. A) Effect of verteporfin on cell migration speed on 2D glass substrates. (n = 20 and 25). B) Number of cells in the outer reservoirs of long microchannels with different widths. B) Representative staining images of RUNX2 in hMSCs after moving out of long microchannels (150 μm) with widths of 10 μm and 3 μm on Day 7 (Images for DMSO group were rescaled for presentation). RUNX2, magenta. Scale: 25 μm. C) Quantification of the N/C ratio of RUNX2. (n = 66, 36, 30, 18, 31, and 8). **$P < 0.01$. ****$P < 0.0001$ ns, no significance. Two tailed unpaired t test was used for S9A. Welch's t test was applied for B. Two-way ANOVA with Bonferroni's post-hoc test was applied for D.

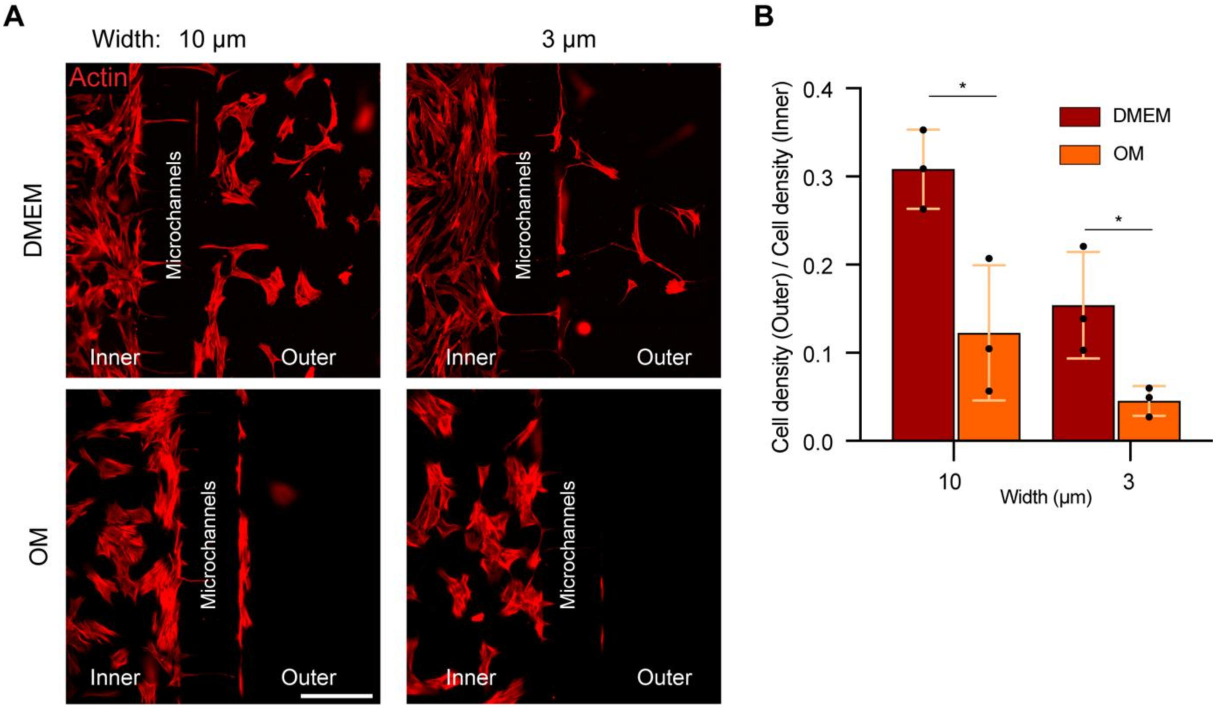

Figure S10. Narrow microchannels do not select for cells with osteogenic potential. A) Representative images of actin staining demonstrating that cells with osteogenic potential did not migrate actively through long confinement. Scale bar, 150 μm. B) The ratio of cell density of outer reservoir and inner reservoir for long microchannels with different widths (n = 3 for each condition). DMEM represents cell expansion media and OM represents osteogenic differentiation media). Two-tailed unpaired t test was used for B. Data are mean ± SD. *$P < 0.001$.

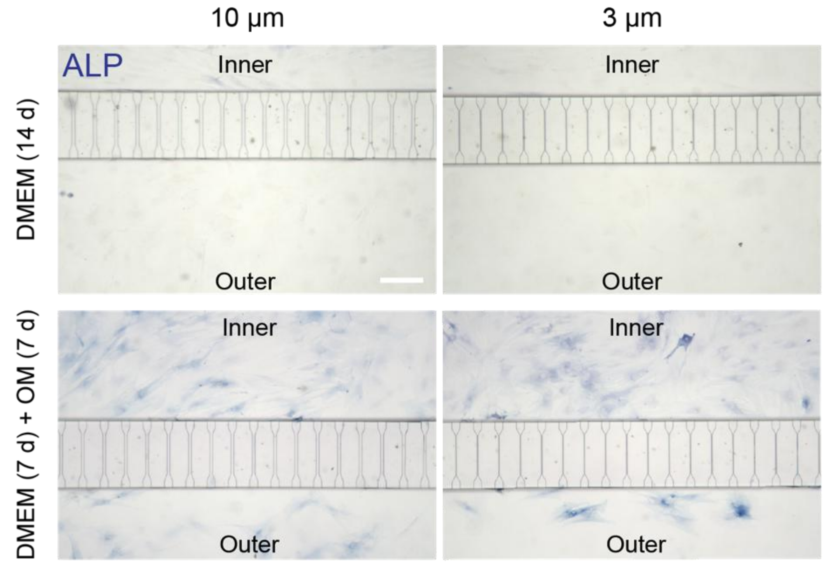

Figure S11. ALP staining in chips on day 14. Cells cultured in chips were maintained under two different conditions: (1) expansion media (DMEM) for 14 days, or (2) expansion media for 7 days followed by osteogenic differentiation media (OM) for an additional 7 days. Scale: 150 μm.

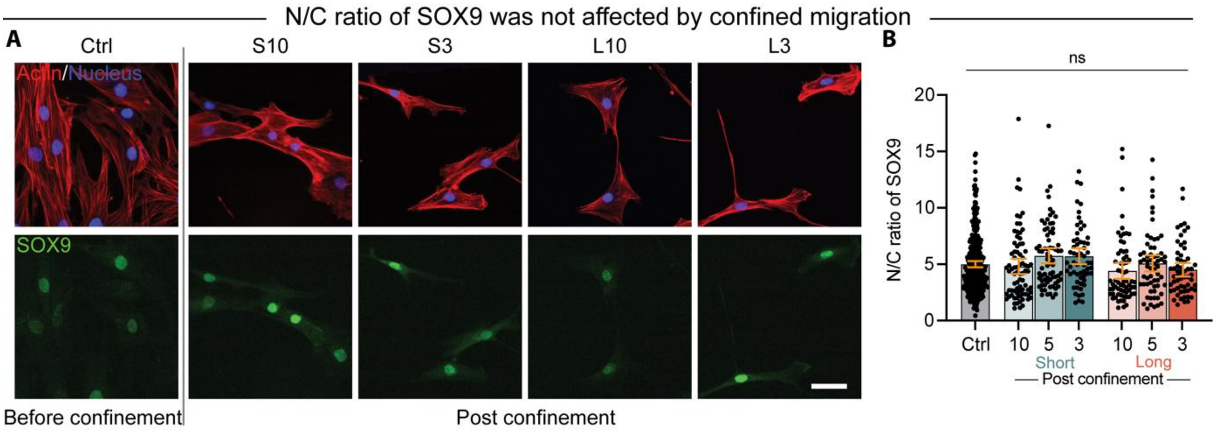

Figure S12. N/C ratio of SOX9 was not affected by confined migration. A) Representative images of hMSCs in regions post 10 µm-wide long microchannel region and 3 µm-wide long microchannel on day 7. SOX9, green; Actin, red; Nucleus, blue. Scale bar: 50 µm. B) Quantification of the N/C ratio of SOX9 in cells before and after migrating through different microchannels on Day 7. (n = 351, 78, 72, 59, 66, 62, and 59). Brown-Forsythe and Welch ANOVA with Games-Howell post-hoc test was applied for B. ns, no significant difference.

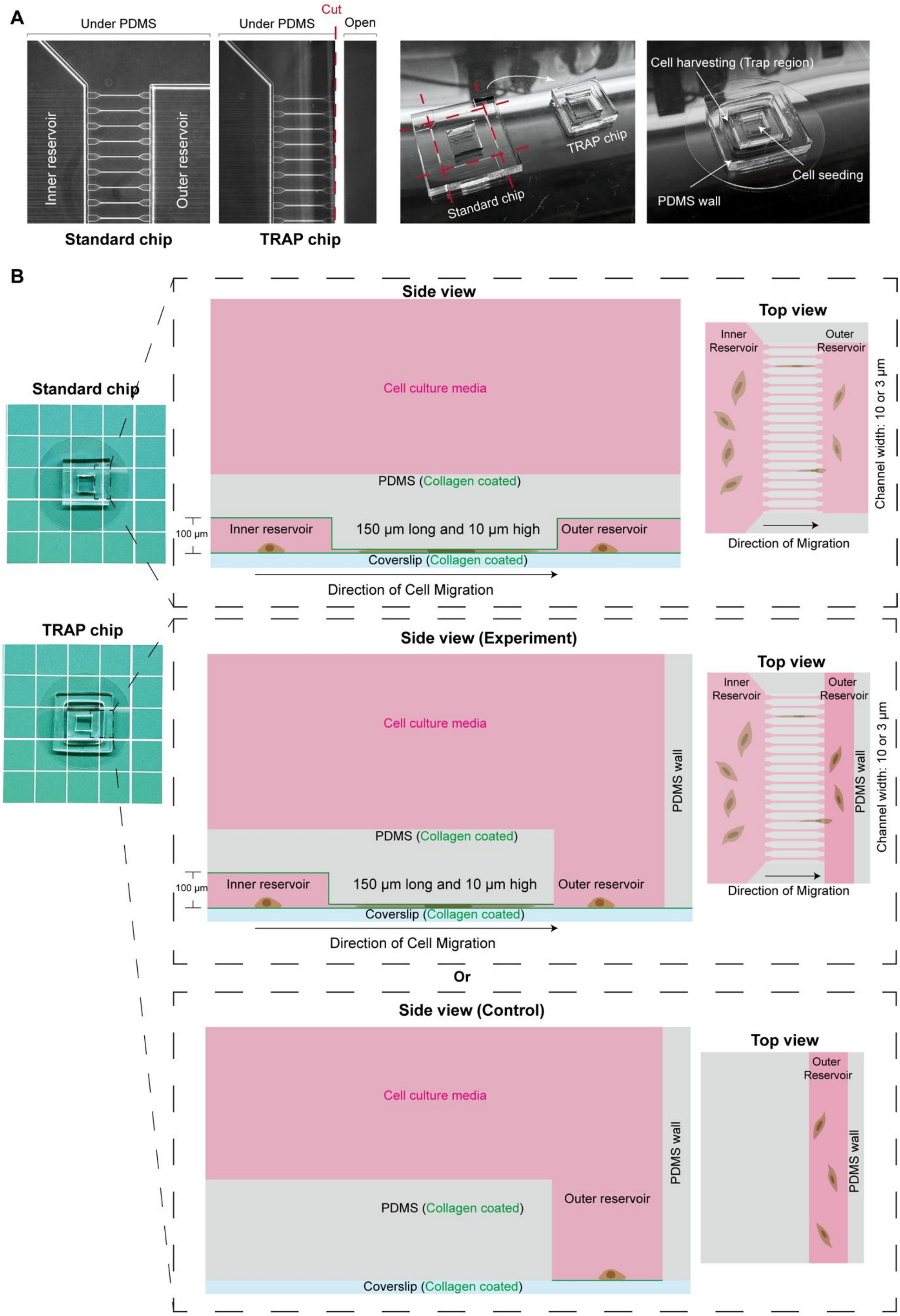
A
Under PDMS
Under PDMS
Cut
Open
Inner reservoir
Outer reservoir
Standard chip
TRAP chip
TRAP chip
Standard chip
Cell harvesting (Trap region)
Cell seeding
PDMS wall
B
Standard chip
TRAP chip
Side view
Cell culture media
PDMS (Collagen coated)
100 µm
Inner reservoir
150 µm long and 10 µm high
Outer reservoir
Coverslip (Collagen coated)
Direction of Cell Migration
Top view
Inner Reservoir
Outer Reservoir
Channel width: 10 or 3 µm
Direction of Migration
Side view (Experiment)
Cell culture media
PDMS wall
PDMS (Collagen coated)
100 µm
Inner reservoir
150 µm long and 10 µm high
Outer reservoir
Coverslip (Collagen coated)
Direction of Cell Migration
Top view
Inner Reservoir
Outer Reservoir
PDMS wall
Channel width: 10 or 3 µm
Direction of Migration
Or
Side view (Control)
Cell culture media
PDMS wall
Outer reservoir
PDMS (Collagen coated)
Coverslip (Collagen coated)
Top view
Outer Reservoir
PDMS wall

Figure S13. Comparison between standard and TRAP microchannel chips. A) Images showing structural differences between standard and TRAP chips, highlighting the absence of PDMS ceiling in the outer reservoir and the presence of a trapping wall in the TRAP chip. B) Schematic representations of the standard and TRAP chips, illustrating differences in microchannel design and functionality.

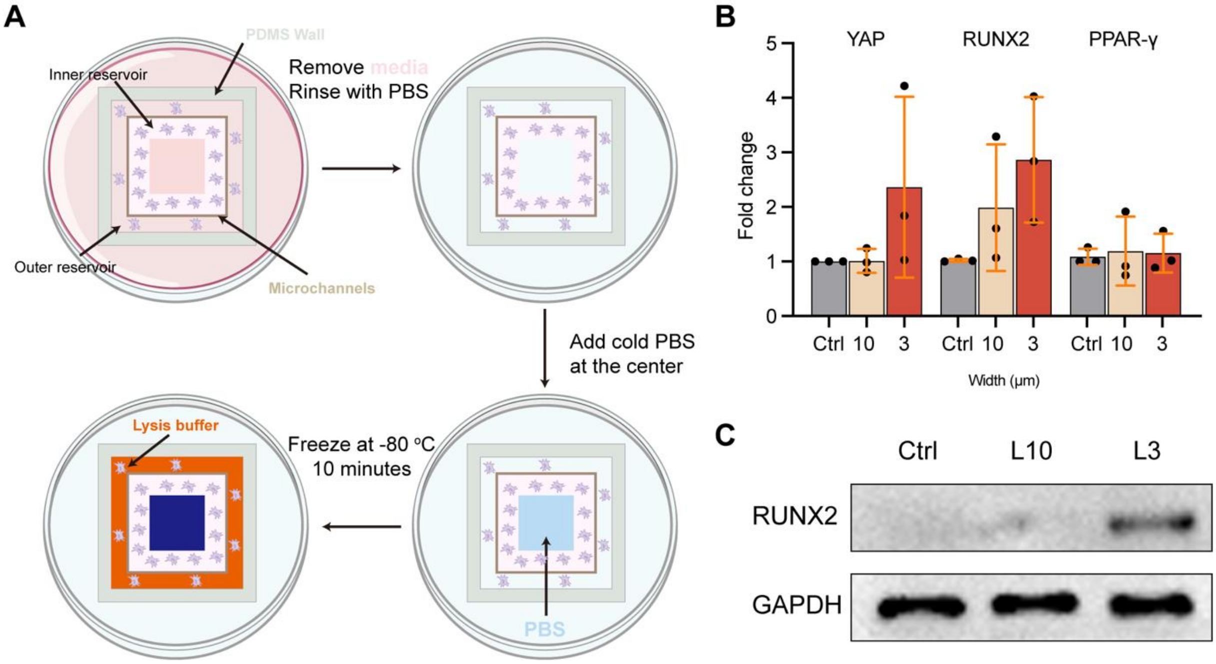

Figure S14. TRAP chip supports collection of cells post-confinement for molecular analysis. A) Illustrations of cell lysate collection steps. B) Quantification of YAP, RUNX2, and PPAR-γ mRNA expression via qPCR (n = 3). C) Representative WB images of RUNX2 and GAPDH protein expression. One-way ANOVA with Tukey's post-hoc test was applied for B. Data are mean ± SD.

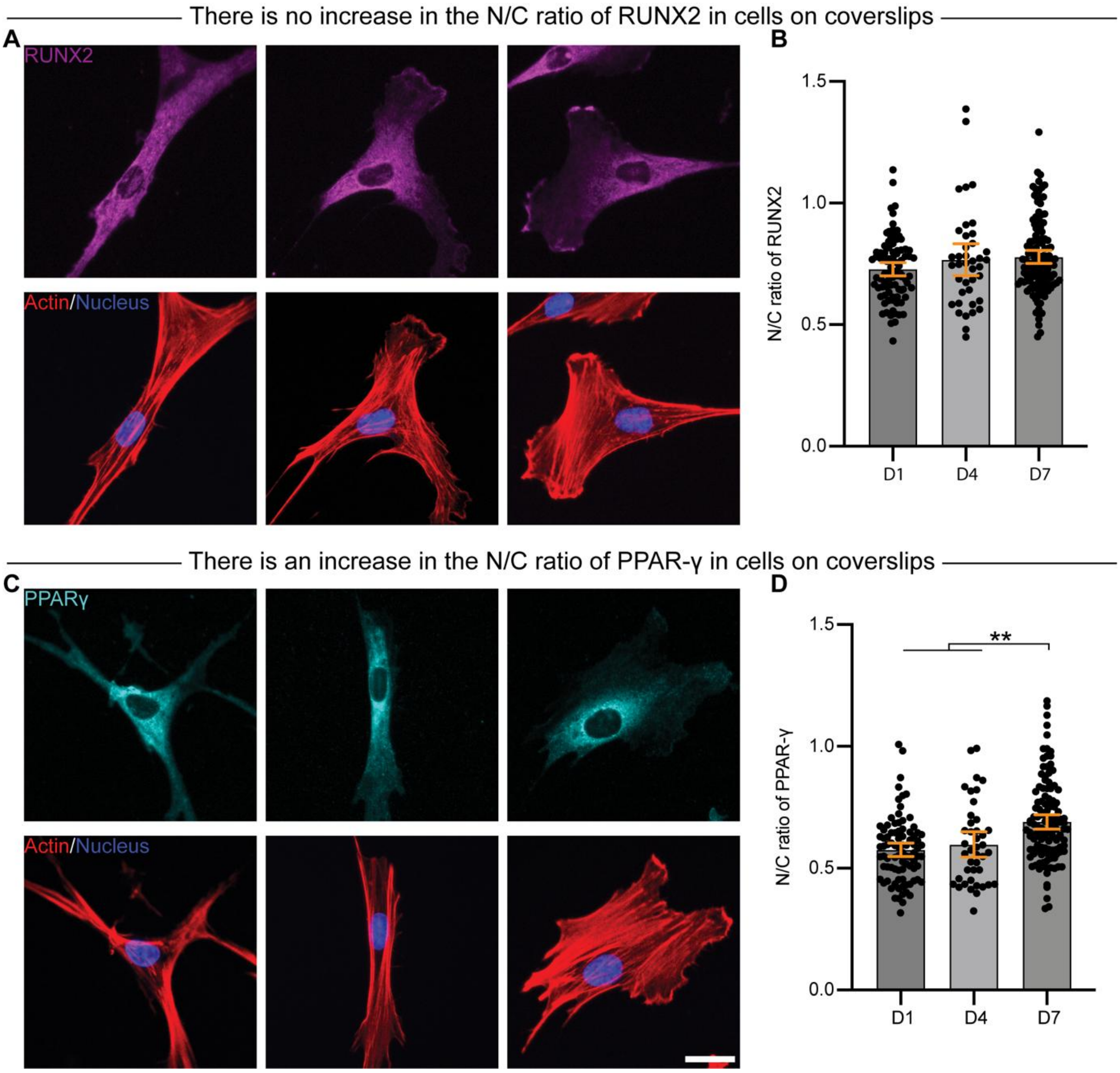

Figure S15. Low cell density did not drive stem cell osteogenesis. A-B) Representative images A) and quantification of N/C ratio of RUNX2 B) of hMSCs after being cultured on coverslips for 7 days. RUNX2, magenta; Actin, red; Nucleus, blue. Scale bar: 25 µm. C-D) Representative images C) and quantification of N/C ratio of PPAR-γ D) of hMSCs after being cultured on coverslips for 7 days. PPAR-γ, cyan; Actin, red; Nucleus, blue. (n = 88, 40 and 124 for B,D). Scale bar: 25 µm. One-way ANOVA with Bonferroni's post-hoc test was used for B and D. ** $P < 0.01$.

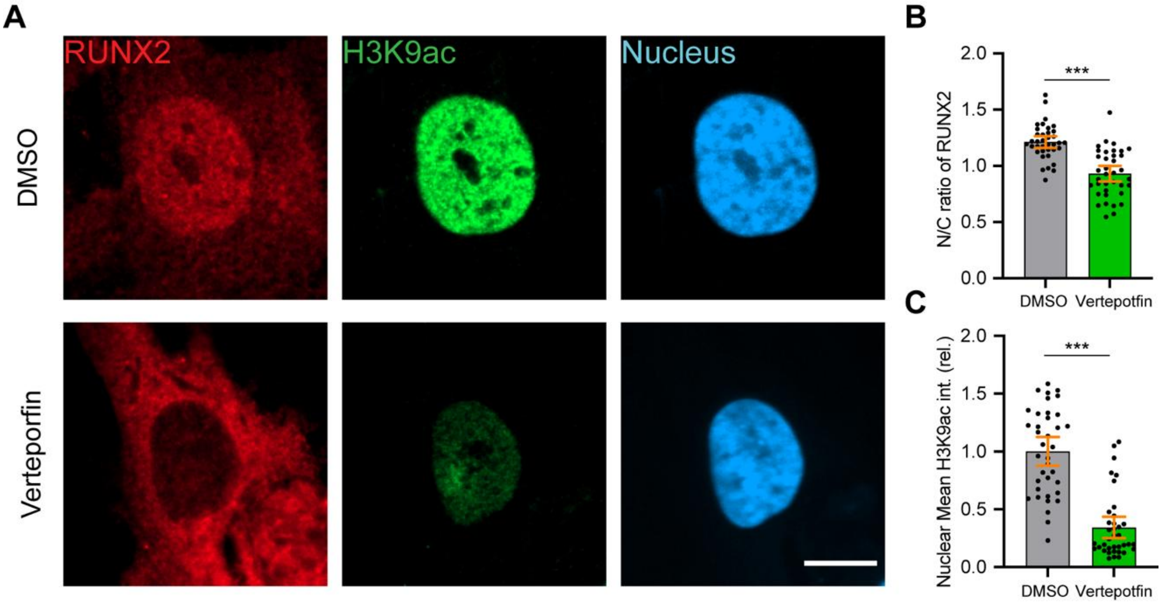

Figure S16. YAP regulates H3K9ac and osteogenesis. A) Representative staining images of RUNX2 and H3K9ac in hMSCs with or without YAP inhibitor on Day 7. RUNX2, red; H3K9ac, green; Nucleus, Blue. Scale bar: 10 μm. B-C) Quantification of the N/C ratio of RUNX2 (B) and nuclear mean intensity of H3K9ac (C) in cells treated with or without YAP inhibitor. (n = 37 and 37). Two-tailed unpaired t test was used for B while Two-tailed Manny-Whitney test was applied for C. ***P< 0.001.

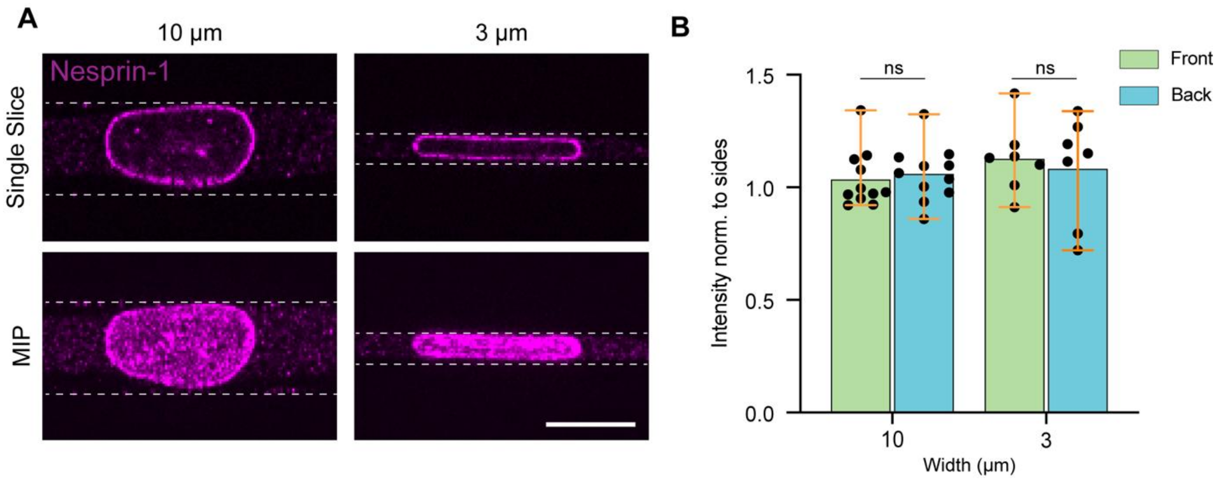

Figure S17. Nesprin positioning under long microchannels. A) Representative images demonstrating nesprin-1 positioning under different degrees of confinement. Scale bar: 10 µm. B) Quantification of the nesprin-1 intensity at cell front and back normalized to the intensity at the sides. n = 11 for 10 µm-wide microchannels and n = 7 for 3 µm-wide microchannels. Two-tailed unpaired test was used for B.

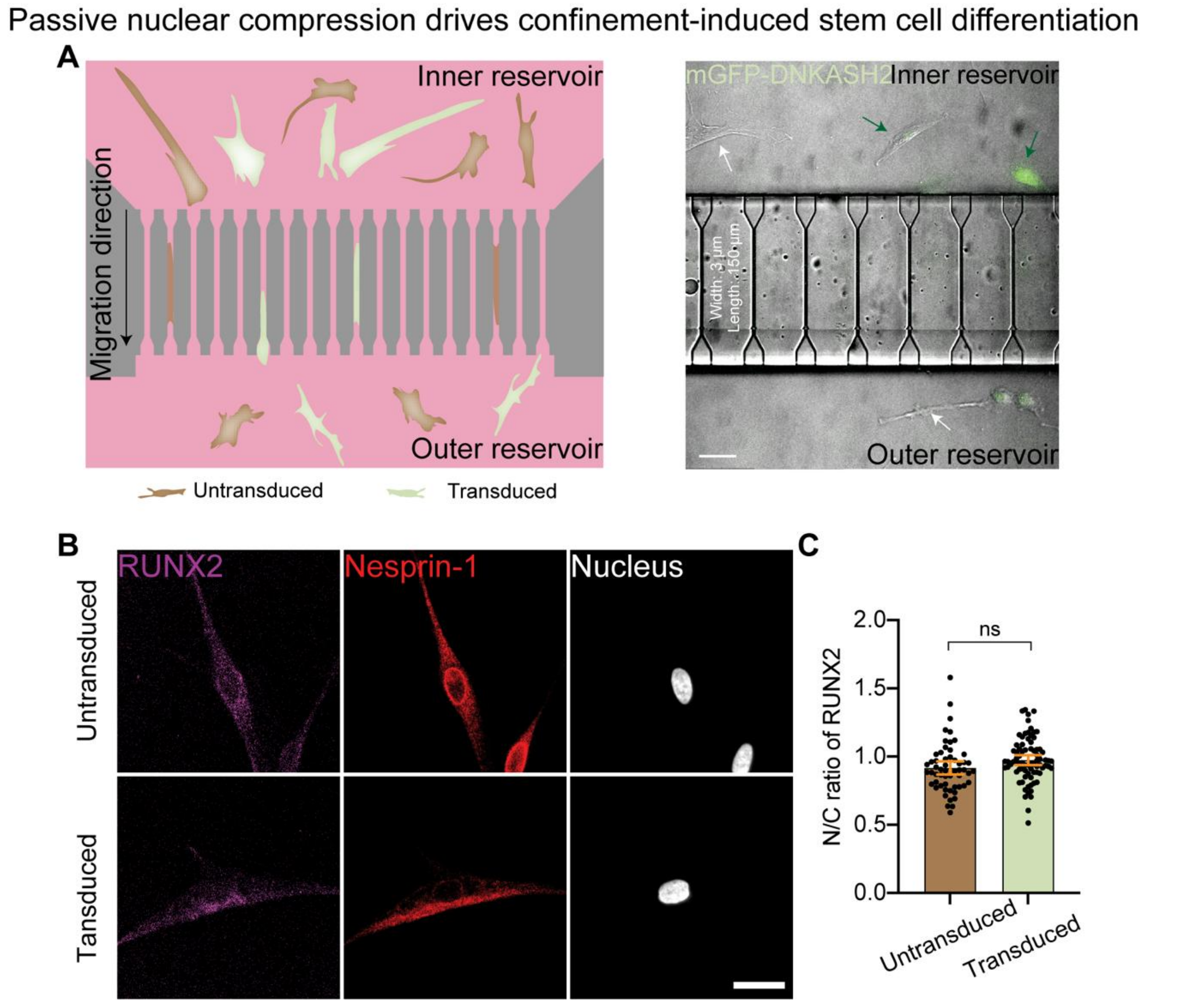

Figure S18. Passive nuclear compression drives confinement-induced stem cell differentiation. A) Schematic (left) of coculture of hMSCs that were successfully transduced (green arrows) and untransduced (white arrows) with mGFP-DNKASH2 after lentiviral transduction in chip and phase contrast image merge with GFP channel (right). Scale bar: 150 µm. B) Representative images of untransduced and transduced hMSCs in regions post 3 µm-wide long microchannel on day 7. RUNX2, magenta; Nesprin-1, red; Nucleus, gray. Scale bar: 25 µm. C) Quantification of N/C ratio of RUNX2 in untransduced and transduced hMSCs in regions post 3 µm-wide long microchannel on day 7. (n = 56 and 79). Unpaired Student T-test was applied for C. ns, no significant difference. Error bars represent 95% confidence intervals.
